# Efficacy and Safety of Lidocam Topical Gel (4% Lidocaine—0.3% Meloxicam) for Pain and Inflammation Management during Castration and Tail Docking in Piglets

**DOI:** 10.3390/ani14060930

**Published:** 2024-03-17

**Authors:** Denis Nagel, Brenda Ralston, Andrea Hanson, Les Burwash, Heather Matheson-Bird, Barbara Olson, Crystal Schatz, Merle Olson

**Affiliations:** 1 Alberta Veterinary Laboratories Ltd., Calgary, AB T2C 5N6, Canada; denis.nagel@avetlabs.com (D.N.); barb.olson@avetlabs.com (B.O.); 2 Applied Research Team, Lakeland College, Vermilion, AB T9X 1K5, Canada; brenda.ralston@lakelandcollege.ca (B.R.); andrea.hanson@lakelandcollege.ca (A.H.); 3 Alberta Agriculture, Airdrie AB T4A 0C3, Canada; aburwash@telus.net; 4 Community Engagements, UCVM, University of Calgary, Calgary, AB T2N 1N4, Canada; heather.mathesonbird@ucalgary.ca; 5 Chinook Contract Research Ltd., Airdrie, AB T4A 0C3, Canada; crystal.schatz@ccr01.com

**Keywords:** meloxicam, lidocaine, topical, piglets, castration, tail docking, pain

## Abstract

**Simple Summary:**

In most high-income countries, piglets undergo certain elective surgical procedures such as castration and tail docking to eliminate behaviors that are associated with mortality and the condemnation of animals and meat. It has been well established that both castration and tail docking are both painful, yet there are limited products for addressing short-term and long-term pain. Lidocam™ Topical Gel (LTG) (4% lidocaine and 0.3% meloxicam) was developed to address the animal welfare and production requirements of the swine industry. The studies described in this paper show that applications of LTG to the scrotal area and tail base act to control the pain associated with these surgical procedure. The studies also demonstrate that LTG was able to control pain and inflammation at the surgical sites for at least 24 h after application. This was demonstrated by using established physiological and behavioral markers for surgical pain: plasma cortisol and substance P, vocalization during procedure, electrocutaneous stimulation of surgical site, and body weight gain. LTG is potentially an effective product for controlling pain and inflammation for use in castration and tail docking in piglets.

**Abstract:**

(1) Background: It has been well established that castration and tail docking are both painful during and following the procedure, yet there are limited convenient and effective products to address both short-term and long-term pain. Lidocam Topical Gel (LTG) (4% lidocaine and 0.3% meloxicam) was developed to address industry needs for an effective and safe product to address animal welfare concerns regarding castration and tail docking in piglets. (2) Methods: Study 1: Male piglets aged 4–8 days of age were treated with LTG (n = 30) or a control gel (n = 30). Approximately 30 min after application of the gel, the piglets were surgically castrated and tail docked. The efficacy of pain control during the surgical procedures and post-procedure (24 h) pain and inflammation control were evaluated using both behavioral and physiological measurements. Study 2: Meloxicam residue depletion following LTG treatment was followed for 28 days. Study 3: Clinical and pathological safety were evaluated in five groups of eight piglets receiving LTG with: (1) no treatment, (2) nominal topical dose, (3) two times the nominal topical dose, (4) three times the nominal topical dose, and 5) one times the nominal topical dose and 2 mL of LTG by oral gavage daily for 3 days. (3) Results: LTG-treated piglets had a significant reduction in electrocutaneous stimulation response before the procedures and 4 and 24 h post-procedures. Stress vocalization intensity and duration were less in piglets receiving LTG during the surgical procedures. Plasma cortisol and substance P were significantly lower in LTG-treated piglets 3 h after castration and tail docking. The weight and average daily gain were significantly increased in piglets receiving LTG. LTG did not interfere with wound healing or cause irritation at the application sites. There were no abnormal clinical or pathological findings associated with the use of LTG at three times the nominal dose given daily for three days. As meloxicam persisted in the application site tissue, a slaughter withdrawal time of 24 days was determined. (4) Conclusions: When applied to the skin 30 min before castration and tail docking, LTG is effective in surgical pain control and provides post-surgical pain control for up to 24 h. LTG is safe for use in piglets and provides an acceptable withdrawal time for commercial use. LTG is a potentially effective product for commercial use for piglet castration and tail docking.

## 1. Introduction

In Canada and in most pork-producing countries, piglets undergo certain elective surgical procedures, and codes of practice have been developed for castration and tail docking [1,2,3,4,5,6,7,8]. Castration is performed to eliminate boar taint and improve the quality of meat [7,8,9,10]. Castration reduces aggressive behavior in post-pubertal male pigs and undesirable pregnancy in co-housed females [7,8,9,10]. Tail docking is performed to reduce tail chewing, which leads to infections, abscesses, morbidity, and mortality, as well as the condemnation of animals and meat [11,12,13,14,15].

It has been well established that both castration and tail docking are painful [16,17,18,19,20,21,22,23,24,25] and that age does not impact an animal’s response to pain, yet there are limited options for providing pain control prior to and after castration and tail docking [26,27,28,29,30,31,32,33,34,35,36,37]. The codes of practice in most countries require the use of anesthetics for the castration procedure and post-operative analgesia of piglets of all ages [1,2,3,4,5,6,7,8]. It is also recognized that the procedures are painful and that this pain persists for at least 24 h and up to 4 days [26,27,28,29,30,31,32,33,34,35,36,37]. However, there are no single treatments that address both pain control at the time of the surgical procedure and post-operatively [26,27,28,29,30,31,32,33].

General anesthetics have been effectively used in piglets for surgical procedures [29,30], but there is a risk of mortality and they are impractical in the industry. Local anesthetic injections (lidocaine HCL) have been shown to provide short-term pain control for castration and tail docking procedures [31,32,33,34,35]. However, injections with lidocaine in the scrotum/testes and as a local subcutaneous block have many issues which make them impractical: (1) lidocaine proves only short-term pain control (approximately 2 h) [31,32,33,34,35]; (2) lidocaine hydrochloride is an acidic solution and is painful when injected subcutaneously [34,35,36]; (3) lidocaine hydrochloride causes tissue damage and delays wound healing [34,35,36]; (4) the time from injection to the surgical procedure is too long for practical applications; (5) there is a significant occupational workplace hazard when using injections of lidocaine; (6) piglets are susceptible to lidocaine hydrochloride toxicity following injection [34]; (7) in some jurisdictions, lidocaine hydrochloride injections should be delivered by a veterinarian who is trained in their use, which is not practical for producers; (8) in some jurisdictions, lidocaine hydrochloride is not labelled for castration and/or tail docking in piglets; and (9) lidocaine use is restricted to cutaneous or epilesional routes in some jurisdictions for residue reasons (European public MRL assessment report (EPMAR) for lidocaine (porcine) EMA/CVMP/393160/2020).

Analgesics (primarily NSAIDS) provided by injections have been shown to be beneficial for piglets undergoing surgical procedures such as castration and tail docking [26,37,38,39,40]; however, they do not provide pain control during these surgical procedures. A major point of resistance to the use of NSAIDS is the requirement to deliver the active agent by injection, as piglets already receive numerous injections (iron and vaccines). NSAIDS can also induce local tissue irritation when injected, which is described on most product labels [41].

Lidocam Topical Gel (LTG) was developed by Alberta Veterinary Laboratories Ltd. (AVL) to address the animal welfare and production requirements of the swine industry. LTG contains 4% lidocaine (as a non-ionic base, without hydrochloric acid) and 0.3% meloxicam as active agents, as well as a penetrating agent (*N*-methyl-2-pyrrolidone) that permits the rapid penetration of lidocaine and meloxicam through the dermis into the subcutaneous space [42,43]. LTG, therefore, has the potential to provide both short-term local anesthesia and long-term control of pain and inflammation [42,43]. The product also contains a blue dye (brilliant blue) to identify treated animals. This product is intended to be practical for swine producers to mitigate pain and reduce inflammation for castration and tail docking in piglets. The objectives of the study described in this paper were (1) to evaluate the efficacy of LTG to relieve pain at the time of the castration and tail docking procedures and control pain and inflammation for at least 24 h post-surgery, (2) to measure meloxicam tissue residues following treatment in order to establish slaughter withdrawal times, and (3) to evaluate the safety of LTG when applied to the skin of male and female piglets.

## 2. Materials and Methods

### 2.1. Efficacy for Pain and Inflammation Control

#### 2.1.1. Animals

The number of animals involved in this study was the minimum number required to generate meaningful and statistically significant results based on power calculations using three variables (electrocutaneous stimulation response, stress vocalization, and plasma cortisol), using alpha = 0.05 and power value of 0.80. The procedures were designed to avoid or minimize discomfort, distress, and pain to the animals. Pre-study approval (Number BVR15-03A) by an Investigational Animal Care and Use Committee (Alberta Agriculture, Airdrie) was obtained. Healthy male Genesis–Red Duroc cross piglets (n = 60, age 4–8 days, 1.64 to 4.3 kg) were obtained from 12 healthy sows in a commercial herd at the same study site where the study was conducted. There was no cross-fostering of piglets, and they were returned to their dam immediately after each data collection. The piglets were distinguished by two forms of identification (ear tattoo and ink (Sharpie) marking on their back, left thorax, and right thorax) which bore the same number and corresponded to a general description of the animal in the study record.

#### 2.1.2. Treatment

The control and treatment (LTG) gels were indistinguishable, as each treatment gel contained brilliant blue dye to blind the treatments to the study participants. Treatment A contained LTG (4% lidocaine base and 0.3% meloxicam, Alberta Veterinary Laboratories Ltd., Calgary, AB, Canada) in a gel matrix. Treatment B contained only the gel matrix without lidocaine or meloxicam. In total, 1 mL of each product was withdrawn into a 1 mL syringe labelled as Treatment A or Treatment B. The syringe contents were deposited over the scrotum area (1 mL) and another syringe content was deposited over the tail base (1 mL). Each identified syringe was weighed before and after use to determine the weight of the product applied to the area. The times of the treatments were also recorded.

#### 2.1.3. Castration and Tail Docking

Castration was carried out by making an initial horizontal incision in the scrotum with a scalpel, after which, the testicles were removed by tearing the spermatic cords [30]. Tail docking was performed using side cutters (blunt cutting method), removing all but approximately 1 cm of the tail. The castration and tail docking times were recorded for each animal.

#### 2.1.4. Blood Collection and Analysis

Blood (1–2 mL) was collected in 3 mL EDTA tubes (BD Vacutainer, Becton Dickinson, Franklin Lakes, NJ, USA) with aprotinin (Sigma-Aldrich, Oakville, ON, Canada) by an anterior vena cava venipuncture on day 0 (time of physical examination, −1 h), t = 3 h, and t = 24 h, post-surgical procedures. The blood was separated and frozen for cortisol and substance P (SP) analyses within 2 h of collection. Cortisol and substance P were analyzed using validated assay kits (EIA Cortisol Kit, Cayman Chemical and Substance P ELISA kit, Enzo Life Sciences, Farmingdale, NY, USA).

#### 2.1.5. Body Weight

The piglets were weighed as part of their pre-study examination (day 0, approximate time = −1 h). They were weighed again on day 6 (7 days after surgical procedures) and day 13 (14 days after surgical procedures).

#### 2.1.6. Wound Healing

The castration and tail docking wounds were assessed with a caliper immediately after the surgical procedures, 3 h after the procedures, and on day 1 (24 h), day 6, and day 13 (7 days and 14 days after the surgical procedures). The castration wound width and length and tail docking wound diameter were measured, except on day 0, where only the scrotal wound length was measured, as the width was highly variable and changed with the position of the animal (the wound was not yet organized). The wounds from each site were scored according to the following scoring system: 1 = wound completely healed, 2 = scab covering wound, wound >60% healed, 3 = scab covering wound, wound 30–60% healed, 4 = scab covering wound, wound 10–30% healed, 5 = no bleeding, scab covering wound, wound 0–10% healed, and 6 = bloody wound with no scab.

#### 2.1.7. Wound Inflammation

Inflammation (swelling and hyperemia) was assessed 3 h after the surgical procedures and on day 1 (24 h), day 6 (7 days), and day 13 (14 days). Inflammation was scored at each surgical site according to the following inflammation scoring system: 0 = no swelling, 1 = slight (swelling with 1 mm of the wound edge), 2 = moderate (swelling 1–3 mm of the wound edge), and 3 = severe (swelling greater than 3 mm of the wound edge). Hyperemia was scored at each surgical site according to the following hyperemia (tissue redness) scoring system: 0 = no hyperemia, 1 = slight (redness with 1 mm of the wound edge), 2 = moderate (redness 1–3 mm of the wound edge), and 3 = severe (redness greater than 3 mm of the wound edge).

#### 2.1.8. Electro-Stimulation of Skin before Surgical Procedures

Cutaneous stimulation was performed using a peripheral variable output nerve stimulator (Sun Medical Microstimulator), and infant monitoring electrodes were used to stimulate the skin over the incisional area of the piglet’s tail or scrotal area just before performing surgery. Electrocutaneous stimulation has been used to evaluate the efficacy and duration of local anesthetics in humans and animals [44,45,46,47]. Briefly, the piglets were placed on an examination table and loosely restrained by hand. After the piglet relaxed, the electrode probe was placed against the test area. The animals were stimulated with a burst of 4 × 60 mAmp stimuli immediately before the surgical procedure and at 4 h and 24 h post-treatment. A positive response to the stimulus included vocalization (squeal), body movement, and tail movement or a negative response (no reaction). Reactions were rated as 0 = no response; 1 = slight response, moves from side to side and tail flick; 2 = moderate response, moves side to side and tail flick, slight kick or jump, and brief squeal (less than 2 s); or 3 = severe response, moves side to side and tail flick, pronounced kick or jump, and squeal (longer than 2 s).

#### 2.1.9. Behavioural Responses

Behavioural responses were evaluated and described by Sutherland et al. [19]. After the procedures (castration, tail docking, blood collection, and wound evaluations), the piglets were returned to their home pen, which was soundproofed from the procedure area. The behaviour of individual pigs was recorded for 60 min at approximately 90 min after castration and tail docking, and again 4 h after these surgical procedures. Observations included: lying without contact (maintaining a recumbent position and not in contact with other pigs or the sow), lying with contact (maintaining a recumbent position while contacting other pig(s) or the sow), nursing (rhythmic and sustained mechanical manipulation of the mammary of the sow by the pigs before, during, and after nursing), sitting (resting on the caudal part of the body), standing (assuming or maintaining an upright position on extended legs), walking (relatively low-speed locomotion in which propulsive force derives from the action of the legs), and pain-like behaviors (scooting, lying, or standing with a hunched back posture). This gave sufficient time after the blood collection and surgeries for the piglets to adjust to a reintroduction to their home pen with their dam.

#### 2.1.10. Stress Vocalization

Stress vocalization measurement was performed according to previously published and validated procedures [18,47,48,49,50,51,52]. A microphone was used to record vocalizations for up to 30 s during castration and tail docking. Vocalization was not present before the surgical procedure and sufficient time was permitted between the castration and tail docking to allow the piglet to stop vocalization. Vocalization was analyzed using a validated stress-call-monitoring system and software (STREMODO version 1, Research Institute for the Biology of Farm Animals, Dummerstorf, Germany) that has been shown to identify painful vocalizations during castration and tail docking [47,48,49,50,51,52]. Only animals that vocalized or with clear recordings were used for comparative analysis.

#### 2.1.11. Statistical Analysis

Individual animals were used as the experimental unit for all measures. Statistical significance was designated a priori at *p* < 0.05. Statistical analysis was performed using validated Graphpad Software 10.1.2 (Graphpad Software, Boston, MA, USA). For repeated measures data (substance P, cortisol, body weight, behavior responses, and stress vocalization), a two-tailed unpaired students T test was performed. For observations involving scores (electro-cutaneous stimulation response score, wound healing score, and inflammation score), a two-tailed Mann–Whitney non-parametric test was performed. Fisher’s exact test was used to compare proportions (e.g., the proportion of animals responding to electrocutaneous stimulation).

### 2.2. Tissue Residue Depletion

This study was conducted in accordance with the guidance document of the International Cooperation on Harmonisation of Technical Requirements for Registration of Veterinary Medicinal Products (VICH) for the conduct of residue studies to determine withdrawal periods in tissues for pigs (VICH GL48) [53] and OECD Principles of Good Laboratory Procedures [54].

#### 2.2.1. Animals and Treatment

The piglets (Genesis–Duroc cross, 3–8 days of age) were assigned a random number and allocated to treatment group by number and sex (2 males and 2 females in each group). All animals received LTG at the doses of 1 mL topically at the tail base and 1 mL on the scrotal (males) or perineal (females) area once and were euthanized accordingly: Groups 1: 2 days post-treatment.; Group 2: 4 days post-treatment; Group 3: 7 days post-treatment; Group 4: 10 days post-treatment; Group 5: 14 days post-treatment; Group 6: 21 days post-treatment; and Group 7: 28 days post-treatment.

##### 2.2.2. Tissue Analysis of Meloxicam

On days 2, 4, 7, 10, 14, 21, and 28 post-treatment, two (2) male and two (2) female animals/sex (total of 4 animals) were humanely euthanized by captive bolt. From each animal, the liver (whole liver), kidney (both kidneys), muscle (thigh muscles), and skin/fat (from the areas of topical application) were excised and harvested for processing. The samples from each animal were weighed, placed in a labelled polyethylene bag, promptly cooled on crushed wet ice, and, within two hours of slaughter, then placed in frozen storage (−20 °C) within 4 h of collection. The tissue samples were then homogenized under frozen conditions. After homogenization, each entire sample was split into two or three approximately equal-weight aliquots and packaged in labelled HDPE screw cap jars.

##### 2.2.3. Meloxicam Analysis

The processed liver, kidney, muscle, and fat/skin samples received at the testing laboratory were logged in, checked for condition, and rapidly transferred to frozen storage (≤−20 °C) to await analysis. As soon as possible after receipt, the 4 samples were subjected to an in vitro analysis for quantification of meloxicam. The analysis of the tissue samples was performed under VICH GL49(R) guidelines [53,54] in a Canadian Food Inspection (CFIA) Certified Laboratory (Silliker, JR Laboratories, 12-3871 North Fraser Road, Burnaby, BC, Canada). Meloxicam was analyzed in the meat, liver, kidney, and skin/fat with a validated procedure (Canadian Food Inspection Agency, CVDR-M-3025.03) using liquid chromatography (LC) and mass spectroscopy detection (MS) [55]. The limit of quantification (LOQ) was 0.5 µg/kg (0.5 ppb). The analytical methods were fully documented and validated a priori.

##### 2.2.4. Statistical Analysis

Descriptive statistics (mean, standard deviation, and minimum/maximum value) were used to study the pig population involved (in terms of age and body weight) and the tissue residue of meloxicam. The calculation of the withdrawal period was based upon statistical methods using accepted, basic pharmacokinetic principles and commercial software (Tool1.1551; Joint FAO/WHO Expert Committee on Food Additives). The upper limit of tolerance interval was 99%, with a 95% confidence interval for Canadian withdrawal calculations, while a tolerance interval at 95% with a 95% confidence interval for was used for European withdrawal calculations. The Canadian (Maximum Residue Limits) MRLs for the edible tissues of swine are Muscle: 20 µg/kg; Liver: 60 µg/kg; Kidney: 200 µg/kg; and Skin/fat: 20 µg/kg, while the MRLs for European edible tissues of swine are: Muscle: 20 µg/kg; Liver: 65 µg/kg; and Kidney: 65 µg/kg.

### 2.3. Target Animal Safety Study

This study was conducted in accordance with the guidance document of the International Cooperation on Harmonisation of Technical Requirements for Registration of Veterinary Medicinal Products (VICH) for target animal safety (TAS) GL43 [56]. The study deviated from the guidance by changing the daily dose in the treatment groups from nominal dose (1 mL per application site), 3 times the nominal dose, and 5 times the nominal dose to nominal dose, 2 times the nominal dose, and 3 times the nominal dose. This deviation was justified, as no more than 3 times the nominal dose could be applied without significant product loss.

#### 2.3.1. Animals

Healthy male Genesis–Red Duroc cross piglets (n = 60, age 4–8 days, 1.64 to 4.3 kg) were obtained from 12 healthy sows in a commercial herd at the same study site where the study was conducted. There was no cross-fostering of the piglets, and they were returned to the dam immediately after each data collection. The piglets were distinguished by two forms of identification (ear tattoo and ink (Sharpie) marking on their back and left and right thorax) which bore the same number and corresponded to a general description of the animal in the study record. The piglets were weighed and randomly allocated (using random number table) to 5 treatment groups of 8 animals (4 males and 4 females), first by body weight (heaviest to lightest) and then by sex. Unallocated piglets of each sex were returned to their dam and not used in the study.

#### 2.3.2. Treatment

The control and LTG gels were indistinguishable, as each treatment gel contained a dye (brilliant blue) to blind the treatments to the study participants. For male piglets, the syringe contents were deposited over the scrotum area and another syringe content was deposited over the tail base. For female piglets, the syringe contents were deposited over the perineal area (between the rectum and the vagina) and another syringe content was deposited over the tail base. Each individually identified syringe was weighed before and after use to determine the weight of the product applied to the area. The times of the treatments were also recorded. The 5 different treatment groups were:

Group 1 consisted of 8 animals (4 males and 4 females) who received LTG at the dose of 1 mL on the tail base and 1 mL on the scrotal (male) or perineal (female) area, administered daily for three (3) consecutive days then euthanized at 48 h after the last treatment.

Group 2 consisted of 8 animals (4 males and 4 females) who received LTG at the dose of 2 mL on the tail base and 2 mL on the scrotal (male) or perineal (female) area, administered daily for three (3) consecutive days then euthanized at 48 h after the last treatment.

Group 3 consisted of 8 animals (4 males and 4 females) who received LTG at the dose of 3 mL on the tail base and 3 mL on the scrotal (male) or perineal (female) area to be administered daily for three (3) consecutive days then euthanized at 48 h after the last treatment.

Group 4 consisted of 8 animals (4 males and 4 females) who received LTG at the dose of 1 mL on the tail base, 1 mL on the scrotal (male) or perineal (female) area, and 2 mL orally to be administered daily for three (3) consecutive days then euthanized at 48 h after the last treatment.

Group 5 (Control) consisted of 8 animals (4 males and 4 females) who received topical gel (without active) at the dose of 1 mL on the tail base and 1 mL on the scrotal (male) or perineal (female) area to be administered daily for three (3) consecutive days then euthanized at 48 h after the last treatment.

#### 2.3.3. Blood and Urine Collection

On days 0, 1, 2, and 4, blood samples (1–2 mL in EDTA tubes) were collected from the anterior vena cava and the time was recorded. On the same day, these samples were sent to a veterinary diagnostic laboratory (IDEXX laboratories, Calgary Alberta) for comprehensive hematology and chemistry. A urine sample was also collected for urinalysis on day 4 (at the time of necropsy). The blood samples were successfully collected from all animals except for one, which died during the blood collection. At necropsy, at the end of the study, there was no evidence of significant trauma to the anterior vena cava because of multiple blood collections.

#### 2.3.4. Haematology

WBC, RBC, Hgb, HCT, erythrocyte indices (MCV, MCH, and MCHC), WBC differential, platelet count, smear evaluation for RBC and WBC blood morphology and parasite screen, review of abnormal cells, reticulocyte count and absolute reticulocyte count, PT, PTT, and quantitative fibrinogen.

#### 2.3.5. Blood Chemistry

Albumin, albumin/globulin ratio, alkaline phosphatase, ALT (SGPT), AST (SGOT), bicarbonate, direct bilirubin, indirect bilirubin, total bilirubin, BUN, BUN/creatinine ratio, calcium, chloride, cholesterol, CK, creatinine, globulin, glucose, phosphorus, potassium, sodium, sodium/potassium ratio, and total protein.

#### 2.3.6. Urinalysis

Volume, color, clarity, specific gravity, pH, protein, glucose, ketones, urobilinogen, bilirubin, blood, WBC, RBC, bacteria, EPI cell, mucus, casts, and crystals.

#### 2.3.7. Observations

Physical examinations were performed on day 0 and day 4, and health assessments were conducted once daily on days 1, 2, and 3, where general appearance, behavior, attitude, feeding behavior, and appetite were assessed. Findings were recorded as “normal” or “abnormal”. The tail base and scrotal area were observed prior to treatment on each day and on day 4 before necropsy, where the degree of irritation was recorded.

#### 2.3.8. Electrocardiograph (ECG)

An ECG sample (15 to 30 s recording) was performed on all animals on day 0 (before treatment, time of physical examination, and 1 h after treatment on day 0, 1, and 2. One hour post treatment is believed to be the optimum time for cardiac lidocaine toxicity, when the drug is absorbed systemically. The QT, PR, QRS, and QQ intervals and heart rates were determined from the ECG recording. ECGs were performed using an Avatar recording system (EEG Solutions Inc., Calgary, AB, Canada), which downloaded recordings to a microchip and transmitted real-time recordings to a tablet computer.

#### 2.3.9. Gross and Microscopic Pathology

On day 4, all animals were humanely euthanized after a physical examination using a non-penetrating captive-bolt stunner, including body weight. A complete gross examination of pathologic changes was performed on all animals. The following tissues (where applicable based on gender) were collected, processed, and packaged in 10% neutral buffered formalin: skin and subcutaneous tissues at the application sites (tail base, scrotum (males), and perineal area (females)), mesenteric lymph nodes, adrenal gland, lung, liver, kidneys, heart (left ventricular wall), stomach, duodenum, jejunum, ileum, cecum, and colon. Histopathology was performed by a board-certified veterinary pathologist who was blinded to the treatment groups (Animal Pathology Services, Edmonton, AB, Canada).

## 3. Results

### 3.1. Study 1: Efficacy for Pain and Inflammation Control

#### 3.1.1. Drug Administration and Castration/Tail Docking

The treatments were performed without complications. The castration and tail docking procedures were also performed without complications. Control or treatment (LTG) gel was provided to each animal at 29–67 min before the time of castration/tail docking. The mean time from treatment to castration or tail docking was 39 ± 9 min.

#### 3.1.2. Electrocutaneous Stimulation of Scrotum and Tail

##### Electrostimulation of Skin Prior to Castration and Tail Docking

Electrocutaneous stimulation was performed on the tail and scrotal surgical sites just prior to performing the castration and tail docking (pre-procedures). At the pre-procedure point in time, lidocaine acted as a topical anesthetic. The results from the pre-procedures are provided in Table 1. At the pre-procedure time (but post-treatment administration), the sensitivity response scores were significantly less in the LTG-treated animals than in the controls at both the tail and scrotal sites (*p* < 0.0001). The proportion of animals not responding to electrocutaneous stimulation was greater in the LTG-treated animals than in the control animals (*p* < 0.0001).

##### Electrocutaneous Stimulation of Skin Surrounding Castration and Tail Docking Wounds at 4 and 24 h Post-Treatment

The results of the electrocutaneous stimulation performed on the tail and scrotal surgical wound sites 4 h and 24 h after performing the tail docking and castration procedures are provided in Table 1. At times 4 and 24 h, lidocaine was not acting as a topical anesthetic, but meloxicam was acting as an analgesic and anti-inflammatory agent in the sensitized tissues. For both time points, the response scores in the LTG-treated animals were significantly less than the response scores in the control animals at both the tail and scrotal sites (*p* < 0.001). The proportion of animals not responding to electrocutaneous stimulation was also significantly greater in the LTG-treated animals than the control animals (*p* < 0.0001).

#### 3.1.3. Stress Vocalization Tail Docking

Stress vocalization (stress call = frequency > 1000 Hz; normal call = frequency < 1000 Hz) was measured during tail docking using a high-resolution microphone and (STREMODO version 1, Research Institute for the Biology of Farm Animals, Dummerstorf, Germany). The percentage of vocalization that was stressful and the duration of the stress vocalization were recorded and are provided in Table 2. The percentage of stress vocalization (percentage of the total call that was at the frequency associated with stress calls) and duration of stress vocalization (duration of the stress calls during the painful procedure (seconds)) were significantly greater in the control animals compared to the LTG-treated animals (*p* = 0.0254, 0.0317). The number of piglets with stress calls (N) during tail docking was also less in the LTG-treated piglets (*p* < 0.05). This was not observed during the castration procedures.

#### 3.1.4. Body Weight

The body weight and weight gain measurements on days 0 (pre-treatment), day 6, and day 13 are provided in Table 3. There was no significant difference in body weights and weight gain between the treatment groups at the pre-treatment weighing (*p* = 0.983). The was a trend for LTG to see an increased weight (*p* = 0.227) and weight gain (*p* = 0.052) on day 6. However, the piglets receiving LTG had a significantly greater weight and weight gain at 14 days post-treatment (*p* = 0.0385 and 0.006, respectively).

#### 3.1.5. Plasma Physiological Markers

##### Blood Collection

Blood (1–2 mL) was collected by an anterior vena cava venipuncture on day 0 (time of physical examination, approximately −1 h), t = 3 h, and t = 24 h post-procedures. One piglet died while blood was being collected from hemorrhage of the anterior vena cava at the 3 h collection time. There were no other complications associated with blood collection. Blood collection was attempted from all piglets. When blood was not collected on the first attempt, venipuncture sample collection was abandoned to avoid injuring the piglet.

##### Plasma Cortisol

The values obtained for plasma cortisol concentration are consistent with those previously published (10 to 400 ηg/mL, depending on the stress and pain levels of the piglets [18,19,20,39,47,57,58,59,60]. The plasma cortisol values (ηg/mL) were natural log-transformed before analysis and the plasma cortisol levels pre-procedures and at times 3 and 24 h post-procedures are provided in Table 4. There was no difference in plasma cortisol at the pre-treatment time (*p* = 0.4411). The mean plasma cortisol levels were significantly increased in the control animals (*p* = 0.0150) at time 3 h, but not at 24 h post-procedures (*p* = 0.1689). There were no differences in the changes in cortisol levels from baseline (time −1 h) to 3 h and baseline to 24 h post-procedures between the LTG-treated and control piglets (*p* = 0.3629 and 0.7475, respectively) (Table 4).

##### Plasma Substance P

The plasma substance P concentrations were consistent with those previously published (50 to 800 ηg/mL, depending on the stress and pain levels of the piglets [57]). The plasma substance P levels (ηg/mL) were natural log-transformed before analysis. The plasma substance P levels pre-castration and at 3 and 24 h post-castration are provided in Table 4. Plasma substance P could not be measured in eight samples due to components within the plasma that interfered with the assay. The plasma substance P levels were not significantly different between treatment groups at the pre-treatment time (*p* = 0.8777). The plasma substance P levels were significantly decreased in the LTG-treated compared to the control piglets at 3 h (*p* **=** 0.0001), but not at 24 h (*p* = 0.9498) post-castration/tail docking. The difference between the pre-treatment and post-treatment substance *p* values was significantly lower in the LTG-treated animals at time 3 h (*p* = 0.0001), but not at 24 h (*p* = 0.3111) between treatment groups.

#### 3.1.6. Visual Behaviour Responses to Castration and Tail Docking

The piglets were observed for 30 min at 1.5 h and 4 h after castration and tail docking in their farrowing crate pen with the presence of female litter mates. At 1.5 h, the mean ± SD and total (T) minutes of lying without contact: (control: 5.8 ± 4.9 min, T = 40.5; LTG: 3.0 ± 1.0, T = 21.0); lying with contact: (control: 17.7 ± 6.9, T = 495; LTG: 18.8 ± 5.9, T = 565); nursing: (control: 8.0 ± 5.5, T = 175.5; LTG: 7.7 ± 5.7, T = 191.5); sitting: (control: 5 ± 0, T = 5; LTG: 0 ± 0, T = 0); standing: (control: 5.4 ± 6.7, T = 107; LTG: 3.3 ± 2.9, T = 73); and walking: (control: 5.3 ± 6.2, T = 74; LTG: 3.1 ± 2.1, T = 49). There were no painful behaviors observed in either group. At 4 h, the mean ± SD and total (T) minutes of lying without contact: (control: 5.5 ± 7.6, T = 33; LTG: 2.8 ± 3.0, T = 34); lying with contact: (control: 18.4 ± 6.9, T = 514; LTG: 19.2 ± 4.3, T = 538); nursing: (control: 9.9 ± 7.3, T = 277.5; LTG: 9.5 ± 6.5, T = 238.5); sitting: (control: 3.0 ± 1.4, T = 6; LTG: 5.0 ± 0, T = 5); standing: (control: 2.0 ± 1.3, T = 19.5; LTG: 2.2 ± 1.7, T = 21.5); and walking: (control: 2.0 ± 0.9, T = 20; LTG: 2.0 ± 1.0, T = 18). There were no painful behaviors observed in either group. There was no difference (*p* > 0.05) in the total times or mean behaviours times between the control or LTG-Treated groups at both the 1.5 and 4 h observation periods.

#### 3.1.7. Wound Dimension and Healing/Inflammation Scores

Following castration (day 0), the wound lengths (mm) for the control piglets were 12.8 ± 3.6 (right) and 13.4 ± 2.6 (left), and for the LTG-treated piglets were 12.9 ± 3.6 (right) and 12.1 ± 2.4 (left). The diameters and lengths were not different (*p* > 0.05). The diameters (mm) of the tail wounds following tail docking were 7.5 ± 1.4 (control) and 7.4 ± 1.1 (LTG). The castration wounds were completely healed in 4 of 58 (6.9%) controls and 4 of 60 (6.6%) LTG-treated animals by day 6, and 100% of wounds were completely healed in both treatment groups by day 13. There was no difference in the castration wound dimension and scores between the control- and LTG-treated animals at 1, 7, and 14 days after castration (*p* > 0.05). None of the tail docking wounds were healed by day 6, but on day 13, 13/29 (45%) of the control and 14/30 (47%) of the LTG-treated tail wounds were completely healed. The diameter (mm) also did not differ between the control- (6.9 ± 1.2) and LTG-treated (6.8 ± 1.0) animals. There was no difference in the tail wound dimension and scores between the control- and LTG-treated animals at 1, 7, and 14 days after castration (*p* > 0.05).

There was no or minimal inflammation associated with the scrotal and tail wounds immediately (30 min post-treatment) and approximately 3 h following the surgical procedures. There was no or minimal inflammation associated with the scrotal and tail wounds 24 h, 7 days, and 14 days post-surgical procedures in both the control- and LTG-treated animals. There was no significant difference in mean wound swelling or hyperemia scores between the LTG-treated and control animals (*p* > 0.05).

Most tail wounds were closed by day 13. There was no difference in the wound healing scores and dimensions between the control- and LTG-treated animals 1, 7, and 14 days after castration (*p* > 0.05).

### 3.2. Study 2: Tissue Residue Depletion

#### 3.2.1. Meloxicam Tissue Concentration (Liver, Kidney, Muscle, Skin/Fat)

The concentrations of meloxicam in the liver, kidney, muscle, and skin/fat are provided in Table 5. On day 28, the meloxicam residue was only measured in the skin/fat from the LTG application site, as the liver, kidney, and muscle concentrations were below level of detection on day 14 and 21. It should be noted that the skin/subcutaneous fat meloxicam concentrations were above the effective serum concentration (700 ηg/mL and 200 ηg/mL) on days 2 and 4. This is a reflection of the long duration provided by topical meloxicam.

#### 3.2.2. Withdrawal Time

The withdrawal time calculation is provided in (Supplementary File S1).

##### Liver

The meloxicam concentrations in the liver were below the Maximum Residue Limits (MRLs) of 60 ηg/g and 65 ηg/g at 2 days post-treatment. The withdrawal periods were calculated to be 6.8 days (Canada) and 3.9 days (Europe).

##### Kidney

The meloxicam concentrations in the kidney were below the MRLs of 65 ηg/g and 200 ηg/g at 2 days post-treatment. The withdrawal periods were calculated to be 6.8 days (Canada) and 6.8 days (Europe).

##### Muscle

The meloxicam concentrations in the muscle were below the MRL of 20 ηg/g at 4 days post-treatment. The withdrawal periods were calculated to be 11.6 days (Canada) and 8.7 days (Europe).

##### Skin/Subcutaneous Fat (Application Site)

The meloxicam concentrations in the skin were below the MRL of 20 ηg/g at further than 21 days post-treatment. The withdrawal period was calculated to be 23.2 days (Canada). There is no MRL for meloxicam in skin/fat in Europe.

### 3.3. Study 3: Target Animal Safety

#### 3.3.1. Observations and Clinical Examinations

All animals were healthy at the time they were enrolled into the study on day 0. All animals remained healthy following treatment, ECG recording, and blood collection, with the exceptions of one animal in Group 2 that died following blood collection on day 1 (trauma from blood collection) and one animal from Group 1 which had trauma to the mandibular area obtained from the sow stepping on the piglet. All other piglets were clinically normal at the time of necropsy on day 4. There was no significant difference in body weight (day 0 and day 4) and body weight gain (day 4) among the treatment groups (*p* > 0.05).

#### 3.3.2. Electrocardiogram Recordings

**Figure 1:**
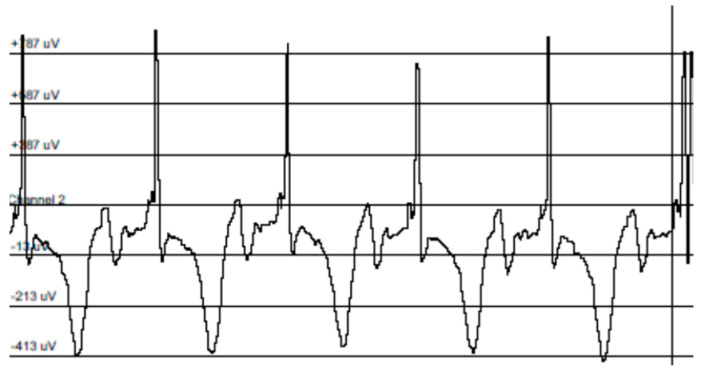
Typical electrocardiogram of a healthy piglet used to measure QT, PR, QRS, and QQ intervals and heart rates.

A typical electrocardogram is provided in Figure 1.

Electrocardiograms were analyzed by measuring the QT, PR, QRS, and QQ intervals and heart rates from the recordings. The electrocardiographic data are summarized in Table 6. The QT, PR, QRS, and QQ intervals and heart rates were normal for neonatal piglets and there was no difference in the QT, PR, QRS, and QQ intervals and heart rates among the treatment groups.

#### 3.3.3. Clinical Chemistry and Hematology

The clinical chemistry and hematology results are provided as (Supplementary File S2), which also includes the clinical pathologist reports (Idexx, Calgary, AB, Canada). No diagnostically significant differences were observed between the control and treatment groups, or between the different treatment groups. Consistent, significant changes suggestive of renal or hepatic disease which would reflect NSAID toxicosis were not evident.

#### 3.3.4. Gross and Histological Pathology

There were no gross pathological changes in any animals in any group, and the pathology report is provided in (Supplementary File S2). There were also no histopathological patterns or clustering of lesions that would suggest an association with a particular test group or groups and not another group, so it was concluded that, under the conditions of the experimental protocol, there were no adverse microscopic anatomic effects of the test article.

## 4. Discussion

Based on the electrocutaneous stimulation and stress vocalization data in this study, it was demonstrated that the scrotal and tail base skin were desensitized to stimulation thirty (30) minutes after the application of the LTG gel. Both electrocutaneous stimulation and stress vocalization have been shown to be effective methods for evaluating the efficacy of local anesthetics and topical analgesics in animals and humans [8,14,44,45,46]. To be effective, the topical anesthetic agent must traverse the skin’s superficial layers and affect the nerve endings within the dermis and subcutaneous tissues. The thickness of the stratum corneum and the acid dissociation constant (pKa) of the anesthetic agent determine how well the topical agent can penetrate the stratum corneum [43,46,61,62,63]. Lidocaine base has a pKa of 7.8, which is like skin [43,61,62,63]. The lidocaine base is unionized and suited to penetrate the stratum corneum and the dermis [61,62,63]. The N-methyl-2-pyrrolidone excipient is a well-established carrier and acts to solubilize the lidocaine and assist in penetration throughout the dermis into the subcutaneous space [42]. Piglet skin is also very thin (like an eyelid), which permits the rapid and effective penetration of lidocaine. Although commercial topical lidocaine (as the ionized hydrochloride form) has been used in humans for the local anesthesia of mucosal surfaces (e.g., oral) and the dermis [43,64,65,66,67], they have not consistently been effective. Topical products are effective for the local anesthesia of mucosal surfaces, but are often not effective for transdermal use in humans and animals [19,44,68,69]. Based upon the electrocutaneous stimulation and stress vocalization results, the efficacy of LTG appears to provide effective local anesthesia and may provide a better option than commercial topical gels or sprays for piglet castration and tail docking. LTG uses lidocaine base, while most commercial topical gels and sprays are formulated with lidocaine HCL. The penetration of the skin is influenced by pH and polarity, resulting in a 50-fold difference in skin penetration between lidocaine base and lidocaine hydrochloride [63]. In addition, LTG also contains N-methyl-2 pyrrolidone, which has been shown to promote the skin penetration of pharmaceutical agents [42]. This formulation difference could explain the efficacy differences among topical local anesthetics.

In this study, LTG at 4 and 24 h post-treatment LTG was effective in significantly reducing the response to electrocutaneous stimulation of the scrotum and tail base after castration and tail docking. Lidocaine would be present at 4 h, but not at 24 h post-treatment [61,62,63]. The electrocutaneous stimulation of tissues at 4 and 24 h post-treatment also evaluated the efficacy of meloxicam in reducing the hyperalgesia produced by the surgical procedures. Although meloxicam is not a local anesthetic, it was effective in significantly reducing the response to electrocutaneous stimulation of the scrotum and tail base after castration and tail docking. Meloxicam and other NSAIDs have been shown to act to reduce pain and inflammation in humans and animals [69,70,71,72,73]. In some cases, oral meloxicam did not provide a beneficial effect in piglets [74], but the efficacy topical meloxicam has not been previously studied in piglets. Topical meloxicam has the advantage over oral or injectable meloxicam of providing a longer therapeutic duration at local tissues [69]. When LTG was applied to scrotal skin, the tissue concentrations of meloxicam were above the therapeutic levels (0.2 µg/g) for over 3 days, while injection (0.4 mg/kg) provides therapeutic levels fir less than 24 h (Olson et al., unpublished data). Topical meloxicam can provide high local tissue levels without the risk of systemic toxicities that are a concern with NSAIDs.

Serum, plasma, and salivary cortisol concentrations are commonly used as a measurement of pain. This hormone provides a relative indication of the overall noxiousness or stress of an experience. In piglets, significant cortisol differences between NSAID/lidocaine-treated and control animals have been reported [21,24,31,32,38,74,75,76]. In this study, significantly different cortisol values were observed in the LTG-treated piglets at 3 h post-castration/tail docking, but there were no differences at 24 h post-castration/tail docking. This was expected, as cortisol changes are usually seen only within a few hours after a painful or stressful event. This suggests that the beneficial effect of LTG in reducing acute pain and/or stress persists for at least 3 h post-surgical procedure.

Substance P is an 11-amino acid neuropeptide that regulates neurons which are involved in the integration of pain, stress, and anxiety. Substance P has pro-inflammatory effects in immune and epithelial cells and participates in inflammatory diseases of the respiratory, gastrointestinal, and musculoskeletal systems. Substance P has been reported to be measured in two other reports, and differences were not observed between the treatment and control groups [57]. In this study, the substance *p* values were significantly lower in the LTG-treated piglets compared to the control piglets measured 3 h after the combination of the castration and tail docking procedures. In addition, there was a significant difference in the change from baseline (reduction) in the substance *p* values between treatment groups in favor of the LTG-treated piglets 3 h post-surgical procedures. This is supportive information that LTG provides short-term anesthesia and analgesia, however, variations in substance P results are common [57]. This benefit could not be statistically demonstrated at 24 h, probably because of the large variance in the plasma values in both the control and treated animals.

Behavioral observations (lying, standing, and walking) in piglets following castration and tail docking have been studied [20,52,73]. Other behavioral evaluations such as facial expression have also been investigated [52]. Behavioral observations do not appear to be a good method for discriminating pain in piglets, as they usually show no difference between treated and control groups. Like many prey mammals and animals in raised in litters where dominance is important, piglets can effectively disguise pain. It is not surprising that, in this study, where the behavioral observation periods were over 1 h, differences between treatment groups could not be demonstrated.

There was no difference in wound inflammation (swelling and hyperemia) scores between the control- and LTG-treated groups. In all the study piglets, there was very little swelling and hyperemia at all the observation times (3 and 24 h post-castration and tail docking), and for this reason, it was not possible to show differences between groups in this study. However, this study demonstrated that LTG does not cause inflammation or irritation to wound sites.

In a previous study, control animals had less swollen castration wounds compared to animals treated with injectable lidocaine hydrochloride and lidocaine hydrochoride/meloxicam [37], probably because injectable lidocaine is known to be irritating when administered in situ. The use of a topical agent such as LTG rather than a parenteral agent has the additional benefit of not causing irritation and pain at the treatment site.

Wound healing is not frequently evaluated in studies where piglets are castrated and tailed docked. It has been shown that piglets treated with a lidocaine spray had improved healing scores [77]. On the other hand, injections with lidocaine reduced healing [37], presumably due to tissue damage caused by the irritation and necrosis associated with acidic injectable lidocaine hydrochloride [34,36]. In this study, there was no treatment effect on the wound healing scores and wound dimensions at either the castration sites or the tail dock sites. As observed previously, topical lidocaine does not impair wound healing [77], and there is a distinct advantage in the use of LTG topical gel compared to injectable lidocaine in situ.

Reduced body weight gain is an indicator of pain, as piglets in pain do not tend to consume as much milk and have abnormal nursing behaviors. Castration and tail docking have been shown to reduce body weight gains in piglets [32,40,75,76,77,78]. Piglets receiving LTG had a significantly greater weight and weight gain 14 days post-treatment. Some investigators have reported improved body weight gain in castrated/tail docking piglets following treatment with locally injected lidocaine and parenteral meloxicam, while others have not shown body weight improvements [32,40,75]. Decreased weight gain is a measurement of stress and pain in most production animals, and this is especially important in the neonates, where feeding behavior can dramatically influence weight gain and growth. The beneficial effect of LTG on body weight gain reflects its ability to reduce pain post-castration and routine tail docking procedures.

As meloxicam acts peripherally to provide pain and inflammation control, it is desirable to have a high local NSAID concentration and low systemic concentrations. This way, there is less risk of potential toxicological events such as gastric, liver, and kidney damage. The toxicity of meloxicam in humans and different mammalian species has been well-studied in laboratory animals, companion animals, cattle, and pigs [79,80,81,82], and meloxicam has consistently been shown to be safe for piglets and adult pigs [79,80,81]. It has also been shown to be less toxic than other NSAIDs, as it is a specific COX-2 antagonist [82,83,84,85]. This study confirmed the safety of topical 4% lidocaine and 0.3% meloxicam, as there were no safety issues, even in piglets receiving three times the nominal dose daily for three subsequent daily treatments. One of the concerns of topical agents is oral intake from self or pen mate licking. This was addressed in a group that was provided both topical and oral LTG daily for three days, which did not show abnormal clinical signs or pathology.

There are limited literature reports on the toxicity of lidocaine to piglets [35,36,86]. The toxicity data in the literature primarily come from human or laboratory animal data [36,87,88,89]. To date, systemic toxicity has been reported in humans receiving high concentrations (10% lidocaine) of topical local anesthetics over a large skin area [89]. These have been only reported with the use of excessive product over large dermal areas [89]. From the published literature, there was a concern that topical lidocaine might interfere with heart electrophysiological function. In dogs, lidocaine toxicity causes a decrease in QT interval, but no change in PT and heart rate [90]. Newborn piglets receiving intravenous lidocaine did not have any cardiovascular effects, but seizures were induced with high dosages [36]. For this reason, ECGs were performed on pigs following the four treatments. There was no evidence that topical lidocaine at three times the dose affected electrophysiological properties. Based on the safety study, it was confirmed that there are no safety concerns when applying topical lidocaine to the tail and scrotum.

Lidocaine residues in piglets receiving injectable or topical have been evaluated by the Committee for Medicinal Products for Veterinary Use, and it was concluded that the establishment of maximum residue limits for lidocaine in porcines is not necessary for the protection of human (consumer) health [91]. For this reason, lidocaine residue depletion studies were not performed. Maximum Residue Limits (MRL) have been established for meloxicam in pigs in most countries and were the reason for conducting a VICH residue depletion study in piglets [92]. This study also provided valuable pharmacokinetic data, which demonstrated therapeutic levels of meloxicam for 4 days. The study also demonstrated that topical meloxicam results in meloxicam residues in edible tissues (meat, liver, kidney, and skin/fat). A withdrawal time was established for LTG of 24 days, which was based on the extended presence of meloxicam in the scrotal tissue application site. The meloxicam residue was below the MRL in the meat, liver, and kidney within 4 days following application. The 24-day withdrawal time should not impair the use of the LTG product, as piglets are not slaughtered for a considerable time after the withdrawal time.

## 5. Conclusions

Based upon the tissue residue study and the efficacy study, LTG provides NSAID-mediated pain and inflammation control from 15 min to 4 days following application. LTG is also safe for use in piglets, as there is no measurable systemic or local toxicity or irritation. The use of LTG for castration and tail docking also resulted in an increased growth rate for the two weeks following castration. The lack of inflammation after castration/tail docking demonstrated that LTG did no harm when applied to the scrotum or tail base prior to castration/tail docking. Although further research is necessary, LTG is a promising single treatment tool for controlling pain and inflammation associated with piglet castration and tail docking.

## 6. Patents

Topical Compositions for the Control of Pain in Animals, Olson Merle, 16/302,628 (USPTO); 17798858.1 (EPO).

## Figures and Tables

**Table 1:** Electrocutaneous stimulation responses scores at the tail and scrotum at immediate pre-procedures, 4 h post-treatment, and 24 h post-procedures. (0 = no response; 1 = slight response, moves from side to side and tail flick; 2 = moderate response, moves side to side and tail flick, slight kick or jump, or brief squeal (less than 2 s); or 3 = severe response, moves side to side and tail flick, pronounced kick or jump, or squeal (greater than 2 s)).

	Control-Treated Animals						LTG-Treated Animals					
ID	Tail 0 h	Scrotum 0 h	Tail 4 h	Scrotum 4 h	Tail 24 h	Scrotum 24 h	Tail 0 h	Scrotum 0 h	Tail 4 h	Scrotum 4 h	Tail 24 h	Scrotum 24 h
N *	29	29	29	29	29	29	30	30	30	30	30	30
Mean	0.9333	1.500	1.000	1.867	0.7586	1.621	0.1667	0.5667	0.2333	0.6667	0.1000	0.1667
SD	0.6397	0.7311	0.8710	0.9371	0.8724	0.9416	0.3790	0.8172	0.5040	0.8023	0.3051	0.3790
*p*-value							<0.0001	<0.0001	<0.0001	<0.0001	<0.0001	<0.0001
N **	7	4	8	4	14	5	25	19	24	16	27	25
*p*-value							<0.0001	<0.0001	<0.0001	<0.0001	<0.0001	<0.0001

* Number of animals tested; ** Number of animals not responding to stimulation.

**Table 2:** Stress vocalization frequency and duration associated with tail docking and castration.

Stress Vocalization during Tail Docking				
	Control-Treated Piglets		LTG-Treated Piglets	
	% High Frequency Stress Calls **	Duration of Stress Calls (s) ***	% High Frequency Stress Calls	Duration of Stress Calls (s)
N *	21	21	14	14
Mean	56.0	2.300	14.0	1.400
SD	12.0	2.221	10.4	0.999
*p*-value			0.0254	0.0317
**Stress Vocalization during Castration**				
	**Control-Treated Piglets**		**LTG-Treated Piglets**	
	% High Frequency Stress Calls **	Duration of Calls (s) ***	% High Frequency Stress Calls ***	Duration of Calls (s) ***
N *	29	29	28	28
Mean	56.0	12.000	46.5	9.000
SD	12.0	4.420	14.3	4.141
*p*-value			0.0054	0.0443

* N = number of animals where squeals were recorded; ** percentage of the total call that was at the frequency associated with stress calls; and *** duration of the stress calls during the painful procedure (s).

**Table 3:** Piglet body weights and body weight change from day 0 (Kg).

Control-Treated Animals						LTG-Treated Animals					
	Day 0 Wt (kg)	Day 6 Wt (kg)	Day 0–6 Wt Change	Day 13 Wt (kg)	Day 0–13 Wt Change		Day 0 Wt (kg)	Day 6 Wt (kg)	Day 0–6 Wt Change	Day 13 Wt (kg)	Day 0–13 Wt Change
N	30	29	29	29	29	N	30	30	30	30	30
Mean	2.686	3.759	1.055	5.437	2.733	Mean	2.698	3.998	1.299	5.860	3.162
SD	0.6829	0.7283	0.5089	0.7734	0.4742	SD	0.6685	0.7723	0.4307	0.7594	0.4336
*p*-value							0.983	0.2273	0.0512	0.0385	0.0060

**Table 4:** Natural log-transformed plasma cortisol and substance P levels in piglets (ηg/mL) sampled at times -1, 3 and 24 h post-castration and tail docking.

Plasma Cortisol Levels in Piglets (ηg/mL)										
Control-Treated Animals						LTG-Treated Animals				
	T = −1 h	T = 3 h	T = 24 h	Diff T-1:T3	Diff T-1:T24	T = −1 h	T = 3 h	T = 24 h	Diff T-1:T3	Diff T-1:T24
N	29	29	29	29	29	30	30	30	28	28
Mean	5.653	5.931	6.127	0.278	0.4989	5.497	5.597	5.941	0.095	0.4386
SD	0.817	0.536	0.447	0.725	0.8114	0.703	0.494	0.5669	0.788	0.5705
*p*-value						0.4411	0.0150	0.1689	0.3629	0.7475
**Plasma Substance P Levels in Piglets (ηg/mL)**										
**Control-Treated Animals**						**LTG-Treated Animals**				
	**T = −1 h**	**T = 3 h**	**T = 24 h**	**Diff** **T-1:T3**	**Diff** **T-1:T24**	**T = −1 h**	**T = 3 h**	**T = 24 h**	**Diff** **T-1:T3**	**Diff** **T-1:T24**
N *	27	27	25	27	25	25	27	26	25	24
Mean	6.224	7.144	5.777	0.920	−0.868	6.207	6.392	5.759	0.136	−0.445
SD	0.278	0.455	1.351	0.372	1.969	0.525	0.659	0.473	0.630	0.501
*p*-value						0.8777	0.0001	0.9498	<0.001	0.3111

N * = Some sample for substance P plasma determination could not be performed due to assay interference.

**Table 5:** Concentration of meloxicam in liver, kidney, muscle, and skin/fat (ηg/g) after administration of 1 mL of 4% lidocaine/0.3% meloxicam to the scrotum and tail.

Sex	Day	Liver	Kidney	Muscle	Skin/Fat
M	2	32.1	98.1	27.5	2160
M	2	34.5	87.3	87.3	1900
F	2	23.2	34.8	17.9	1020
F	2	22.0	55.0	24.6	580
F	4	6.2	9.1	8.9	450
F	4	4.9	8.3	4.2	646
M	4	4.7	8.5	5.1	359
M	4	5.5	10.6	5.2	59.1
F	7	0.8	8.9	9.1	71.5
F	7	0.8	1.6	0.5	71.6
M	7	3.3	6.8	2.5	97.3
M	7	0.5	1.4	1.7	320
F	10	<0.5	<0.5	1.2	20.2
F	10	1.7	1.9	1.6	41.1
M	10	<0.5	<0.5	1.3	231
M	10	0.7	1.7	2.8	71.7
M	14	<0.5	<0.5	<0.5	21.5
F	14	<0.5	<0.5	1.1	18.8
M	14	<0.5	<0.5	0.8	44.2
F	14	<0.5	<0.5	<0.5	21.3
M	21	<0.5	<0.5	<0.5	4.1
M	21	<0.5	<0.5	<0.5	6.6
F	21	<0.5	<0.5	<0.5	<0.5
F	21	<0.5	<0.5	<0.5	1.4
F	28				<0.5
M	28				<0.5
F	28				<0.5
M	28				<0.5

**Table 6:** QT, PR, QRS, and QQ intervals and heart rate of piglets before and after treatment.

		Group 1 1×	Group 2 2×	Group 3 3×	Group 4 1× + Oral	Group 5 Control	*p* Value
QT Interval (s)							
Day 0 Pre-Treatment	Mean	0.157	0.154	0.149	0.156	0.162	0.8154
	SD	0.006	0.012	0.0147	0.026	0.035	
Day 0 (1 h Post-Treatment)	Mean	0.162	0.157	0.159	0.167	0.174	0.2499
	SD	0.0169	0.0153	0.014	0.013	0.019	
Day 1 (1 h Post-Treatment)	Mean	0.159	0.161	0.161	0.144	0.173	0.0799
	SD	0.015	0.009	0.011	0.029	0.019	
Day 2 (1 h Post-Treatment)	Mean	0.146	0.158	0.154	0.155	0.155	0.7910
	SD	0.019	0.015	0.022	0.019	0.013	
PR Interval (s)							
Day 0 Pre-Treatment	Mean	0.120	0.120	0.115	0.122	0.118	0.4653
	SD	0.008	0.007	0.006	0.011	0.011	
Day 0 (1 h Post-Treatment)	Mean	0.119	0.119	0.114	0.119	0.117	0.7975
	SD	0.008	0.010	0.006	0.007	0.015	
Day 1 (1 h Post-Treatment)	Mean	0.126	0.125	0.123	0.128	0.126	0.7664
	SD	0.009	0.012	0.003	0.007	0.009	
Day 2 (1 h Post-Treatment)	Mean	0.123	0.124	0.119	0.122	0.122	0.9110
	SD	0.009	0.013	0.009	0.007	0.010	
QRS Interval (s)							
Day 0 Pre-Treatment	Mean	0.031	0.031	0.031	0.032	0.033	0.4787
	SD	0.002	0.003	0.002	0.004	0.003	
Day 0 (1 h Post-Treatment)	Mean	0.033	0.029	0.033	0.032	0.032	0.1105
	SD	0.004	0.005	0.003	0.002	0.002	
Day 1 (1 h Post-Treatment)	Mean	0.034	0.034	0.037	0.034	0.033	0.1566
	SD	0.003	0.005	0.004	0.003	0.002	
Day 2 (1 h Post-Treatment)	Mean	0.035	0.036	0.035	0.035	0.032	0.3650
	SD	0.003	0.005	0.002	0.004	0.003	
QQ Interval (s)							
Day 0 Pre-Treatment	Mean	0.393	0.363	0.358	0.378	0.395	0.8195
	SD	0.056	0.017	0.028	0.090	0.135	
Day 0 (1 h Post-Treatment)	Mean	0.385	0.379	0.408	0.387	0.430	0.4875
	SD	0.051	0.036	0.087	0.045	0.082	
Day 1 (1 h Post-Treatment)	Mean	0.370	0.374	0.387	0.388	0.412	0.6269
	SD	0.034	0.031	0.051	0.045	0.093	
Day 2 (1 h Post-Treatment)	Mean	0.373	0.385	0.402	0.397	0.397	0.8944
	SD	0.039	0.036	0.079	0.061	0.083	
Heart Rate (beats/min)							
Day 0 Pre-Treatment	Mean	155	166	169	164	162	0.7941
	SD	19	8	13	27	34	
Day 0 (1 h Post-Treatment)	Mean	158	160	152	157	144	0.6288
	SD	18	16	28	19	27	
Day 1 (1 h Post-Treatment)	Mean	163	161	157	156	151	0.7764
	SD	15	14	19	17	29	
Day 2 (1 h Post-Treatment)	Mean	162	157	154	154	155	0.9134
	SD	16	13	26	19	24	

## Data Availability

The data presented in this study are available on request from the corresponding author. The data are not publicly available due to confidentially of the commercial herd owner.

## Supplementary Materials

The following supporting information can be downloaded at: https://www.mdpi.com/article/10.3390/ani14060930/s1, Supplementary File S1. Calculation of Withdrawal time. Supplementary File S2. Pathology Reports.
