# Supplementary material for: Efficacy and Safety of Lidocam Topical Gel (4% Lidocaine—0.3% Meloxicam) for Pain and Inflammation Management during Castration and Tail Docking in Piglets
Source: Animals (Basel). 2024 Mar 17;14(6):930. doi: 10.3390/ani14060930 (PMC10967310; doi:10.3390/ani14060930)
Supplement: Supplementary file 1 [file animals-14-00930-s001.zip › Supplementary File S2 Pathology Reports.zip!/Clinical Pathology Report.pdf]

Piglets- NSAID study

General comments

1) The reference intervals listed on the printed reports reflect expected values for healthy, adult pigs. Typical values for very young piglets may be well outside of these established adult intervals.
2) SDMA (symmetric dimethylarginine) is a marker for early renal disease in cats and dogs; significance in other species has not been determined. Higher values are expected in dogs and cats <6 months old, but this trend may or may not occur in other species. Recommend disregard this assay in the context of the current study.
3) Presence of nucleated erythrocytes and large numbers of reticulocytes in circulation likely reflects normally active hematopoiesis in view of age of the subjects. Individual variation is expected.
4) Glucose- Mild to moderate elevations may occur with physiologic stress. Low glucose value is a relatively common artifact (utilization of glucose by erythrocytes in unseparated samples).
5) Creatinine- lower values are typically seen in young animals; muscle mass is low in comparison to adults.
6) Phosphorus- typically higher in neonates and growing animals than adults.
7) Calcium- May be higher in normal neonates than in adults of the same species.
8) Protein - Values may be lower in young animals than adults, often due to lower globulin levels. Mild increases in protein levels over the course of the study may reflect volume contraction and/or absorption of globulins from colostrum.
9) ALP- Values above the adult reference interval are expected for young animals, and may reflect normal activity of the bone isoenzyme of ALP related to normal growth.
10) GGT- Levels are typically high in neonatal large animals, resulting from absorption of high levels of GGT in colostrum.
11) Bilirubin- sample hemolysis interferes with measurement.
12) PT and PTT- Very mild deviations from collection protocol (for example inadequate sample volume for the volume of anticoagulant in the tube) can result in values outside of the reference interval.
13) Platelets- low automated platelet count is a common artifact, related to sample clotting with collection or platelet clumping in the sample.
14) WBC: Lymphopenia is a common finding without diagnostic significance, related to physiologic stress. Lymphocytosis can be seen with excitement/epinephrine response or antigenic stimulation.
15) Clotting - Sample clotting typically results in low RBC count and HCT, as well as decreased platelet numbers. Decreased leukocyte counts may occur as well, in an inconsistent manner.

16) USG - Neonates typically are not able to concentrate urine as well as adults. USG is expected to vary widely, reflecting individual variation and differences in water/fluid intake.

17) Urine sediment- Hematuria is often iatrogenic in nature. Bacteria without inflammation typically reflects sample contamination.

BLACK

Glucose: No significant findings. Low values may be artifact.

Urea: There are no diagnostically significant changes evident. Mild variation likely reflects individual/physiologic variation.

Creatinine: Unremarkable findings diagnostically for this group.

Phosphorus: Unremarkable findings diagnostically for this group.

Calcium: Unremarkable findings diagnostically for this group.

Sodium, chloride, bicarbonate: No significant diagnostic findings.

Total protein, albumin, and globulin: Albumin values generally slightly higher at day 4 than day 0, possibly reflecting mild volume contraction. No diagnostically significant changes are evident.

Alanine transaminase (ALT): Values consistent for individuals in this group. No diagnostically significant findings.

Aspartate transaminase (AST): Values consistent for individuals in this group. No diagnostically significant findings.

Alkaline phosphatase (ALP): Values for each individual are relatively consistent. No diagnostically significant changes.

Gamma GT (GGT): Several individuals showed an increase at day 2, possibly related to colostrums absorption. For one piglet increase in GGT was accompanied by icterus (black3); increased concern regarding biliary oriented hepatic lesion.

Bilirubin, serum icterus: Mild elevations of bilirubin in several individuals +/- icterus. For black 7 and 8, this change was evident at the beginning of the study, starting at day zero. For black 3, mild elevation of bilirubin and mild icterus of serum were evident at day 2 and day 4. Possibilities include mild hepatic disease and normal individual variation. Prehepatic icterus (due to mild hemolysis) is possible.

WBC: Leukocyte counts are within reference for all individuals. Stress lymphopenia noted. Neutrophilia suggestive of inflammation is not evident.

PT/PTT: No significant findings. Marginal elevation of several PTT values is noted, most likely artifact.

Urinalysis: Wide variation in USG as expected. Hematuria in one sample (black 2) is suspected to be iatrogenic. Protein may reflect sediment in this sample. For the sample from black 6, trace protein with USG of 1.019 is of uncertain significance -cannot entirely rule out renal disease.

PURPLE

Glucose: Values within reference. No significant findings.

Urea, creatinine, phosphorus, calcium: No diagnostically significant findings.

Sodium, chloride, bicarbonate: Purple 4 has slightly lower chloride than others in the group. May be normal individual variation, or could reflect fluid pooling in the upper gastrointestinal tract. No significant findings otherwise.

Total protein, albumin, and globulin: Albumin values increase mildly from day 0 to day 4, possibly reflecting mild volume contraction. No diagnostically significant changes are evident.

Alanine transaminase (ALT): No diagnostically significant findings.

Aspartate transaminase (AST): Mild elevation (approaching upper end of the reference interval) of AST on day 0 for purple 8 may reflect myocyte injury (CK is mildly elevated) or may be artifact related to sample hemolysis. Value back to baseline by day 1, no clinical concern. Otherwise unremarkable for this group.

Alkaline phosphatase (ALP): No diagnostically significant changes.

GGT: Transient elevation (day 2) is noted for 2 individuals (purple 3 and purple 6), possibly related to colostrum absorption. Unlikely diagnostically significant.

Bilirubin, icterus: Transient elevations of bilirubin with or without concurrent sample icterus are noted for several individuals in this group. Possibilities include normal physiologic variation, mild cholestasis, or prehepatic icterus (hemolytic disease).

WBC: Within reference, no significant concerns.

PT/PTT: Marginal elevations noted in several individuals are likely artifact. Significant elevation for purple 1 on day 4 may be artifact, for example sample clotting leading to consumption of coagulation factors in the sample. Cannot rule out coagulopathy, however this seems unlikely in view of unremarkable results on the previous assays for this individual.

Sodium, chloride, bicarbonate: Blue 4 has mildly decreased chloride values compared to others in the group. May be normal individual variation, or could reflect fluid pooling in the upper gastrointestinal tract. No significant findings otherwise.

Total protein, albumin, globulin: General trend of mild increase in albumin and protein from day 0 to day 4 likely reflects mild volume contraction. No diagnostically significant changes are evident.

Alanine transaminase (ALT): No diagnostically significant findings.

Aspartate transaminase (AST): Unremarkable for this group.

Alkaline phosphatase (ALP): No diagnostically significant changes.

GGT: Transient elevations for several individuals, possibly related to colostrum absorption. Unlikely diagnostically significant.

Bilirubin, icterus: Mild elevations of bilirubin with concurrent sample icterus are noted for blue 7 and blue 8. Possibilities include normal physiologic variation, mild cholestasis, or prehepatic icterus (hemolytic disease).

WBC: Low values may reflect normal variation with species/age or artifact. Blue 5 shows consistently higher WBC and neutrophils than others in the group- possibilities include inflammation, stress and normal individual variation.

PT/PTT: Marginal elevations noted in several individuals are most likely artifact. High PT and PTT for blue 6 and blue 7 on day 0 are attributed to artifact; values are within reference for the remainder of the assays for these individuals.

Urinalysis: No diagnostic concerns.

Summary

No diagnostically significant differences are appreciated between control and treatment groups, or between the different treatment groups.

Consistent, significant changes suggestive of renal or hepatic disease are not evident.

Dr Lily Edwards, DVM, MVSc, Dipl. ACVP
Sept 29 2015
