# Supplementary material for: Efficacy and Safety of Lidocam Topical Gel (4% Lidocaine—0.3% Meloxicam) for Pain and Inflammation Management during Castration and Tail Docking in Piglets
Source: Animals (Basel). 2024 Mar 17;14(6):930. doi: 10.3390/ani14060930 (PMC10967310; doi:10.3390/ani14060930)
Supplement: Supplementary file 1 [file animals-14-00930-s001.zip › Supplementary File S2 Pathology Reports.zip!/Histopathology Report.pdf]

Target Animal Safety Study of AVL's Lidocaine - Meloxicam Topical Gel in Piglets: Histopathology

Animal Pathology Services (APS) Ltd.
18208 Ellerslie Road,
Edmonton,
Alberta T6W 1A5
Study Number 15-177

Bow Valley Research Study BVR-15-02

Sponsor: Alberta Veterinary Laboratories Ltd.,
411 19th Street SE
Calgary, AB T2E 6J7

Final Report
9 November 2015

TABLE OF CONTENTS

Title Page 1
Table of Contents 2
Summary 3
Procedure 4
Results 4
Discussion 28

LIST OF TABLES

Table I: Summary of Histopathologic Findings 5
Table II: Individual Animal data 8

SUMMARY

The objective of this histopathology study was to assess the safety of AVL's Lidocaine-Meloxicam Topical Gel in piglets.

The scope of this histopathology study was limited to examination and reporting on the histopathologic findings in selected organs from study animals according to BVR Protocol BVR 15-02. The study protocol and group assignment of individual animals were provided by the study director, but no other data was received or assessed in preparing this report.

It was concluded that under the experimental conditions employed in BVR Protocol 15-02 there were no adverse microscopic anatomic effects of the test article.

The histopathological examination and preparation of this report were conducted by the undersigned.

P. N. Nation, DVM, PhD, DACVP
Animal Pathology Services (APS) Ltd.

PROCEDURE:

A total of 232 microscope slides of organs from 39 pigs from study BVR 15-02 were received from Bow Valley Research via HSRL. Microscope slides were read and observations recorded for each animal directly into individual animal records in Table 2 below. Following completion of the microscopic examination and recording of findings for individual animals, results were summarized by experimental group in Table 1.

Following completion of the microscopic examination and recording of findings, this report was prepared. The author is aware of the group assignment and sex of each animal but is blinded as to the treatment given each group. As the scope of this report is limited to histopathologic findings, all additional correlations of clinical pathology, necropsy and weight data will be made by the study director.

All slides were found to be well stained and representative of the organs from which they were taken, and therefore were suitable for comparison to similar sections for the same organ from the other animals in the study. Occasional individual tissues were missing but this was infrequent and did not compromise the interpretation of study results.

RESULTS:

The microscopic findings in the individual animals of this study are presented in Table 2 and these are organized and presented by study group in Table 1. Table 2 constitutes the raw data of this study as the findings were entered directly into Table 2 as observations were made. Table 1 presents the summary of histopathologic data and as such, microscopic findings that may have been categorized slightly differently in Table 2 that are essentially the same are grouped together in Table 1 for the sake of clarity.

Table Number: 1
Summary of Histopathologic Findings

Organs / Diagnoses / Modifiers	Group: / Sacrifice:	Blue SN[1]	Purple SN	Red SN	Black SN	Green SN
	Total Animals:	8	7	8	8	8
Kidney						
Within Normal Limits		8	6	8	8	8
Cellular debris, tubules, outer cortex, focal						
Minimal		0	1	0	0	0
Lung						
Within Normal Limits		8	6	7	7	6
Activation, alveolar macrophages, localized						
Minimal		0	1	0	0	0
Infiltrate, macrophages, alveoli and alveolar septae, locally extensive or lobular						
Moderate		0	0	1	1	0
Cellular debris, bronchioles and/or alveoli, focal or locally extensive						
Minimal		0	0	0	1	0
Moderate		0	0	1	0	0
Alveolitis, granulomatous, multifocal to locally extensive						
Mild		0	0	0	0	1
Pneumonia, interstitial, lobular distribution, chronic						
Severe		0	0	0	0	1

[1] Scheduled Necropsy

**Liver**					
Within Normal Limits	2	1	0	2	1
Not Included	0	0	0	0	1
Extramedullary hematopoiesis, sinusoids, multifocal					
Minimal	1	5	4	1	2
Mild	0	0	1	3	3
Moderate	5	1	3	2	1
**Heart**					
Within Normal Limits	8	7	8	8	8
**Stomach**					
Within Normal Limits	7	6	8	7	7
Infiltrate, lymphocytes, lamina propria, multifocal/locally extensive/diffuse					
Minimal	0	2	0	0	1
Mild	0	0	0	1	0
Moderate	1	0	0	0	0
Infiltrate, lymphocytes, muscularis, perivascular, focal/multifocal					
Minimal	0	1	0	0	0
Mild	1	0	0	0	0
Mineral deposits, crypts, lumen, focal					
Minimal	0	0	0	1	0
**Duodenum**					
Within Normal Limits	8	6	8	8	8
Not Included	0	1	0	0	0
**Jejunum**					
Within Normal Limits	8	7	8	8	8

Ileum					
Within Normal Limits	8	7	8	8	8
Cecum					
Within Normal Limits	1	1	1	0	0
Mineralized cellular debris, crypts, lumen, mutifocal					
Minimal	3	2	3	4	3
Mild	4	1	2	2	2
Moderate	0	3	2	2	3
Neutrophils and cellular debris, crypts, lumen, multifocal					
Mild	0	0	1	0	0
Colon					
Within Normal Limits	7	6	8	7	8
Not Included	1	1	0	1	0
Mesenteric Lymph Nodes					
Within Normal Limits	7	7	8	7	8
Not Included	1	0	0	1	0
Skin (tail base)					
Within Normal Limits	7	7	8	8	8
Not Included	1	0	0	0	0
Skin (scrotal)					
Within Normal Limits	7	7	8	8	8
Not Included	1	0	0	0	0

Table Number: 2
Individual Animal Data
1= minimal, 2 = mild, 3 = moderate, 4 = severe

Species: Porcine Animal Number: Blue 1 Sex: F
Group: Blue Fate: (SN) Scheduled Necropsy

Microscopic Findings	
Kidney	Within Normal Limits
Lung	Within Normal Limits
Liver	Extramedullary hematopoiesis, sinusoids, multifocal, moderate
Heart	Within Normal Limits
Stomach	Within Normal Limits
Duodenum	Within Normal Limits
Jejunum	Within Normal Limits
Ileum	Within Normal Limits
Cecum	Mineralized cellular debris, crypts, lumen, mutifocal, minimal
Colon	Not Included
Mesenteric Lymph Nodes	Within Normal Limits
Skin (tail base)	Not Included
Skin (scrotal)	Not Included

Species: Porcine Animal Number: Blue 2 Sex: F
Group: Blue Fate: (SN) Scheduled Necropsy

Microscopic Findings	
Kidney	Within Normal Limits
Lung	Within Normal Limits
Liver	Extramedullary hematopoiesis, sinusoids, multifocal, moderate
Heart	Within Normal Limits
Stomach	Within Normal Limits
Duodenum	Within Normal Limits
Jejunum	Within Normal Limits
Ileum	Within Normal Limits
Cecum	Mineralized cellular debris, crypts, lumen, multifocal, mild
Colon	Within Normal Limits
Mesenteric Lymph Nodes	Within Normal Limits
Skin (tail base)	Within Normal Limits
Skin (scrotal)	Within Normal Limits

Species: Porcine Animal Number: Blue 3 Sex: F
Group: Blue Fate: (SN) Scheduled Necropsy

Microscopic Findings

Kidney	Within Normal Limits
Lung	Within Normal Limits
Liver	Extramedullary hematopoiesis, sinusoids, multifocal, moderate
Heart	Within Normal Limits
Stomach	Within Normal Limits
Duodenum	Within Normal Limits
Jejunum	Within Normal Limits
Ileum	Within Normal Limits
Cecum	Mineralized cellular debris, crypts, lumen, mutifocal, mild
Colon	Within Normal Limits
Mesenteric Lymph Nodes	Within Normal Limits
Skin (tail base)	Within Normal Limits
Skin (scrotal)	Within Normal Limits

Species: Porcine Animal Number: Blue 4 Sex: F
Group: Blue Fate: (SN) Scheduled Necropsy

Microscopic Findings

Kidney	Within Normal Limits
Lung	Within Normal Limits
Liver	Extramedullary hematopoiesis, sinusoids, multifocal, moderate
Heart	Within Normal Limits
Stomach	1. Infiltrate, lymphocytes, lamina propria, multifocal to diffuse, moderate<br>2. infiltrate, lymphocytes, muscularis, perivascular, multifocal, mild
Duodenum	Within Normal Limits
Jejunum	Within Normal Limits
Ileum	Within Normal Limits
Cecum	Mineralized cellular debris, crypts, lumen, mutifocal, minimal
Colon	Within Normal Limits
Mesenteric Lymph Nodes	Within Normal Limits
Skin (tail base)	Within Normal Limits
Skin (scrotal)	Within Normal Limits

Species: Porcine	Animal Number: Blue 5	Sex: M
Group: Blue	Fate: (SN) Scheduled Necropsy	

Microscopic Findings

Kidney	Within Normal Limits
Lung	Within Normal Limits
Liver	Extramedullary hematopoiesis, sinusoids, multifocal, moderate
Heart	Within Normal Limits
Stomach	Within Normal Limits
Duodenum	Within Normal Limits
Jejunum	Within Normal Limits
Ileum	Within Normal Limits
Cecum	Mineralized cellular debris, crypts, lumen, mutifocal, mild
Colon	Muscle and mesentery present but mucosa not in plane of section
Mesenteric Lymph Nodes	Not Included
Skin (tail base)	Within Normal Limits
Skin (scrotal)	Within Normal Limits
Testis	Within Normal Limits for an immature piglet

Species: Porcine	Animal Number: Blue 6	Sex: M
Group: Blue	Fate: (SN) Scheduled Necropsy	

Microscopic Findings

Kidney	Within Normal Limits
Lung	Within Normal Limits
Liver	Extramedullary hematopoiesis, sinusoids, multifocal, minimal
Heart	Within Normal Limits
Stomach	Within Normal Limits
Duodenum	Within Normal Limits
Jejunum	Within Normal Limits
Ileum	Within Normal Limits
Cecum	Mineralized cellular debris, crypts, lumen, mutifocal, minimal
Colon	Within Normal Limits
Mesenteric Lymph Nodes	Within Normal Limits
Skin (tail base)	Within Normal Limits
Skin (scrotal)	Within Normal Limits

Species: Porcine   Animal Number: Blue 7   Sex: M
Group: Blue   Fate: (SN) Scheduled Necropsy

Microscopic Findings	
Kidney	Within Normal Limits
Lung	Within Normal Limits
Liver	Within Normal Limits
Heart	Within Normal Limits
Stomach	Within Normal Limits
Duodenum	Within Normal Limits
Jejunum	Within Normal Limits
Ileum	Within Normal Limits
Cecum	Mineralized cellular debris, crypts, lumen, mutifocal, minimal
Colon	Within Normal Limits
Mesenteric Lymph Nodes	Within Normal Limits
Skin (tail base)	Within Normal Limits
Skin (scrotal)	Within Normal Limits

Species: Porcine   Animal Number: Blue 8   Sex: M
Group: Blue   Fate: (SN) Scheduled Necropsy

Microscopic Findings	
Kidney	Within Normal Limits
Lung	Within Normal Limits
Liver	Within Normal Limits
Heart	Within Normal Limits
Stomach	Within Normal Limits
Duodenum	Within Normal Limits
Jejunum	Within Normal Limits
Ileum	Within Normal Limits
Cecum	Mineralized cellular debris, crypts, lumen, mutifocal, mild
Colon	Within Normal Limits
Mesenteric Lymph Nodes	Within Normal Limits
Skin (tail base)	Within Normal Limits
Skin (scrotal)	Within Normal Limits

Species: Porcine   Animal Number: Purple 1   Sex: F
Group: Purple   Fate: (SN) Scheduled Necropsy

Microscopic Findings	
Kidney	Cellular debris, tubules, outer cortex, focal, minimal
Lung	Within Normal Limits
Liver	Extramedullary hematopoiesis, sinusoids, multifocal, minimal
Heart	Within Normal Limits
Stomach	Within Normal Limits
Duodenum	Within Normal Limits
Jejunum	Within Normal Limits
Ileum	Within Normal Limits
Cecum	Mineralized cellular debris, crypts, lumen, mutifocal, moderate
Colon	Within Normal Limits
Mesenteric Lymph Nodes	Within Normal Limits
Skin (tail base)	Within Normal Limits
Skin (scrotal)	Within Normal Limits

Species: Porcine   Animal Number: Purple 2   Sex: F
Group: Purple   Fate: (SN) Scheduled Necropsy

Microscopic Findings	
Kidney	Within Normal Limits
Lung	Within Normal Limits
Liver	Extramedullary hematopoiesis, sinusoids, multifocal, minimal
Heart	Within Normal Limits
Stomach	Infiltrate, lymphocytes, lamina propria, focal, minimal
Duodenum	Not Included
Jejunum	Within Normal Limits
Ileum	Within Normal Limits
Cecum	Within Normal Limits
Colon	Not Included
Mesenteric Lymph Nodes	Within Normal Limits
Skin (tail base)	Within Normal Limits
Skin (scrotal)	Within Normal Limits

Species: Porcine Animal Number: Purple 3 Sex: F
Group: Purple Fate: (SN) Scheduled Necropsy

Microscopic Findings

Kidney	Within Normal Limits
Lung	Activation, alveolar macrophages, localized, minimal
Liver	Extramedullary hematopoiesis, sinusoids, multifocal, moderate
Heart	Within Normal Limits
Stomach	1. Infiltrate, lymphocytes, lamina propria, multifocal to diffuse, minimal<br>2. infiltrate, lymphocytes, muscularis, perivascular, focal. minimal
Duodenum	Within Normal Limits
Jejunum	Within Normal Limits
Ileum	Within Normal Limits
Cecum	Mineralized cellular debris, crypts, lumen, mutifocal, moderate
Colon	Within Normal Limits
Mesenteric Lymph Nodes	Within Normal Limits
Skin (tail base)	Within Normal Limits
Skin (scrotal)	Within Normal Limits

Species: Porcine Animal Number: Purple 4 Sex: F
Group: Purple Fate: (SN) Scheduled Necropsy

Microscopic Findings

Kidney	Within Normal Limits
Lung	Within Normal Limits
Liver	Extramedullary hematopoiesis, sinusoids, multifocal, minimal
Heart	Within Normal Limits
Stomach	Within Normal Limits
Duodenum	Within Normal Limits
Jejunum	Within Normal Limits
Ileum	Within Normal Limits
Cecum	Mineralized cellular debris, crypts, lumen, mutifocal, moderate
Colon	Within Normal Limits
Mesenteric Lymph Nodes	Within Normal Limits
Skin (tail base)	Within Normal Limits
Skin (scrotal)	Within Normal Limits

Species: Porcine Animal Number: Purple 5 Sex: M
Group: Purple Fate: (SN) Scheduled Necropsy

Microscopic Findings	
Kidney	Within Normal Limits
Lung	Within Normal Limits
Liver	Extramedullary hematopoiesis, sinusoids, multifocal, minimal
Heart	Within Normal Limits
Stomach	Within Normal Limits
Duodenum	Within Normal Limits
Jejunum	Within Normal Limits
Ileum	Within Normal Limits
Cecum	Mineralized cellular debris, crypts, lumen, mutifocal, mild
Colon	Within Normal Limits
Mesenteric Lymph Nodes	Within Normal Limits
Skin (tail base)	Within Normal Limits
Skin (scrotal)	Within Normal Limits

Species: Porcine Animal Number: Purple 6 Sex: M
Group: Purple Fate: (SN) Scheduled Necropsy

Microscopic Findings	
Kidney	Within Normal Limits
Lung	Within Normal Limits
Liver	Within Normal Limits
Heart	Within Normal Limits
Stomach	Within Normal Limits
Duodenum	Within Normal Limits
Jejunum	Within Normal Limits
Ileum	Within Normal Limits
Cecum	Mineralized cellular debris, crypts, lumen, mutifocal, moderate
Colon	Within Normal Limits
Mesenteric Lymph Nodes	Within Normal Limits
Skin (tail base)	Within Normal Limits
Skin (scrotal)	Within Normal Limits

Species: Porcine   Animal Number: Purple 8   Sex: M
Group: Purple   Fate: (SN) Scheduled Necropsy

Microscopic Findings

Kidney	Within Normal Limits
Lung	Within Normal Limits
Liver	Extramedullary hematopoiesis, sinusoids, multifocal, minimal
Heart	Within Normal Limits
Stomach	Within Normal Limits
Duodenum	Within Normal Limits
Jejunum	Within Normal Limits
Ileum	Within Normal Limits
Cecum	Mineralized cellular debris, crypts, lumen, mutifocal, minimal
Colon	Within Normal Limits
Mesenteric Lymph Nodes	Within Normal Limits
Skin (tail base)	Within Normal Limits
Skin (scrotal)	Not Included

Species: Porcine   Animal Number: Red 1   Sex: F
Group: red   Fate: (SN) Scheduled Necropsy

Microscopic Findings

Kidney	Within Normal Limits
Lung	Within Normal Limits
Liver	Extramedullary hematopoiesis, sinusoids, multifocal, moderate
Heart	Within Normal Limits
Stomach	Within Normal Limits
Duodenum	Within Normal Limits
Jejunum	Within Normal Limits
Ileum	Within Normal Limits
Cecum	Mineralized cellular debris, crypts, lumen, mutifocal, minimal
Colon	Within Normal Limits
Mesenteric Lymph Nodes	Within Normal Limits
Skin (tail base)	Within Normal Limits
Skin (scrotal)	Within Normal Limits

Species: Porcine Animal Number: Red 2 Sex: F
Group: Red Fate: (SN) Scheduled Necropsy

Microscopic Findings

Kidney	Within Normal Limits
Lung	Within Normal Limits
Liver	Extramedullary hematopoiesis, sinusoids, multifocal, mild
Heart	Within Normal Limits
Stomach	Within Normal Limits
Duodenum	Within Normal Limits
Jejunum	Within Normal Limits
Ileum	Within Normal Limits
Cecum	Within Normal Limits
Colon	Within Normal Limits
Mesenteric Lymph Nodes	Within Normal Limits
Skin (tail base)	Within Normal Limits
Skin (scrotal)	Within Normal Limits

Species: Porcine Animal Number: Red 3 Sex: F
Group: Red Fate: (SN) Scheduled Necropsy

Microscopic Findings

Kidney	Within Normal Limits
Lung	1. Infiltrate, macrophages, alveoli and alveolar septae, locally extensive, moderate<br>2. cellular debris, alveoli, locally extensive, moderate
Liver	Extramedullary hematopoiesis, sinusoids, multifocal, moderate
Heart	Within Normal Limits
Stomach	Within Normal Limits
Duodenum	Within Normal Limits
Jejunum	Within Normal Limits
Ileum	Within Normal Limits
Cecum	Mineralized cellular debris, crypts, lumen, mutifocal, mild
Colon	Within Normal Limits
Mesenteric Lymph Nodes	Within Normal Limits
Skin (tail base)	Within Normal Limits
Skin (scrotal)	Within Normal Limits

Species: Porcine Animal Number: Red 4 Sex: F
Group: Red Fate: (SN) Scheduled Necropsy

Microscopic Findings	
Kidney	Within Normal Limits
Lung	Within Normal Limits
Liver	Extramedullary hematopoiesis, sinusoids, multifocal, minimal
Heart	Within Normal Limits
Stomach	Within Normal Limits
Duodenum	Within Normal Limits
Jejunum	Within Normal Limits
Ileum	Within Normal Limits
Cecum	Mineralized cellular debris, crypts, lumen, mutifocal, moderate
Colon	Within Normal Limits
Mesenteric Lymph Nodes	Within Normal Limits
Skin (tail base)	Within Normal Limits
Skin (scrotal)	Within Normal Limits

Species: Porcine Animal Number: Red 5 Sex: M
Group: Red Fate: (SN) Scheduled Necropsy

Microscopic Findings	
Kidney	Within Normal Limits
Lung	Within Normal Limits
Liver	Extramedullary hematopoiesis, sinusoids, multifocal, moderate
Heart	Within Normal Limits
Stomach	Within Normal Limits
Duodenum	Within Normal Limits
Jejunum	Within Normal Limits
Ileum	Within Normal Limits
Cecum	1. Mineralized cellular debris, crypts, lumen, mutifocal, minimal<br>2. Neutrophils and cellular debris, crypts, lumen, multifocal, mild
Colon	Within Normal Limits
Mesenteric Lymph Nodes	Within Normal Limits
Skin (tail base)	Within Normal Limits
Skin (scrotal)	Within Normal Limits

Species: Porcine Animal Number: Red 6 Sex: M
Group: Red Fate: (SN) Scheduled Necropsy

Microscopic Findings

Kidney	Within Normal Limits
Lung	Within Normal Limits
Liver	Extramedullary hematopoiesis, sinusoids, multifocal, minimal
Heart	Within Normal Limits
Stomach	Within Normal Limits
Duodenum	Within Normal Limits
Jejunum	Within Normal Limits
Ileum	Within Normal Limits
Cecum	Mineralized cellular debris, crypts, lumen, mutifocal, mild
Colon	Within Normal Limits
Mesenteric Lymph Nodes	Within Normal Limits
Skin (tail base)	Within Normal Limits
Skin (scrotal)	Within Normal Limits

Species: Porcine Animal Number: Red 7 Sex: M
Group: Red Fate: (SN) Scheduled Necropsy

Microscopic Findings

Kidney	Within Normal Limits
Lung	Within Normal Limits
Liver	Extramedullary hematopoiesis, sinusoids, multifocal, mild
Heart	Within Normal Limits
Stomach	Within Normal Limits
Duodenum	Within Normal Limits
Jejunum	Within Normal Limits
Ileum	Within Normal Limits
Cecum	Mineralized cellular debris, crypts, lumen, mutifocal, moderate
Colon	Not Included
Mesenteric Lymph Nodes	Within Normal Limits
Skin (tail base)	Within Normal Limits
Skin (scrotal)	Within Normal Limits

Species: Porcine Animal Number: Red 8 Sex: M
Group: Red Fate: (SN) Scheduled Necropsy

Microscopic Findings

Kidney	Within Normal Limits
Lung	Within Normal Limits
Liver	Extramedullary hematopoiesis, sinusoids, multifocal, minimal
Heart	Within Normal Limits
Stomach	Within Normal Limits
Duodenum	Within Normal Limits
Jejunum	Within Normal Limits
Ileum	Within Normal Limits
Cecum	Mineralized cellular debris, crypts, lumen, mutifocal, minimal
Colon	Within Normal Limits
Mesenteric Lymph Nodes	Within Normal Limits
Skin (tail base)	Within Normal Limits
Skin (scrotal)	Within Normal Limits

Species: Porcine Animal Number: Black 1 Sex: F
Group: Black Fate: (SN) Scheduled Necropsy

Microscopic Findings

Kidney	Within Normal Limits
Lung	1. Infiltrate, mixed mononuclear cells and lymphocytes, alveoli and alveolar septae, lobular, moderate<br>2. Celular debris, alveoli and bronchioles, focal, minimal
Liver	Extramedullary hematopoiesis, sinusoids, multifocal, minimal
Heart	Within Normal Limits
Stomach	Within Normal Limits
Duodenum	Within Normal Limits
Jejunum	Within Normal Limits
Ileum	Within Normal Limits
Cecum	Mineralized cellular debris, crypts, lumen, mutifocal, moderate
Colon	Within Normal Limits
Mesenteric Lymph Nodes	Within Normal Limits
Skin (tail base)	Within Normal Limits
Skin (scrotal)	Within Normal Limits

Species: Porcine Animal Number: Black 2 Sex: F
Group: Black Fate: (SN) Scheduled Necropsy

Microscopic Findings	
Kidney	Within Normal Limits
Lung	Within Normal Limits
Liver	Extramedullary hematopoiesis, sinusoids, multifocal, moderate
Heart	Within Normal Limits
Stomach	Within Normal Limits
Duodenum	Within Normal Limits
Jejunum	Within Normal Limits
Ileum	Within Normal Limits
Cecum	Mineralized cellular debris, crypts, lumen, mutifocal, minimal
Colon	Within Normal Limits
Mesenteric Lymph Nodes	Not Included
Skin (tail base)	Within Normal Limits
Skin (scrotal)	Within Normal Limits

Species: Porcine Animal Number: Black 3 Sex: F
Group: Black Fate: (SN) Scheduled Necropsy

Microscopic Findings	
Kidney	Within Normal Limits
Lung	Within Normal Limits
Liver	Within Normal Limits
Heart	Within Normal Limits
Stomach	Within Normal Limits
Duodenum	Within Normal Limits
Jejunum	Within Normal Limits
Ileum	Within Normal Limits
Cecum	Mineralized cellular debris, crypts, lumen, mutifocal, mild
Colon	Within Normal Limits
Mesenteric Lymph Nodes	Within Normal Limits
Skin (tail base)	Within Normal Limits
Skin (scrotal)	Within Normal Limits

Species: Porcine   Animal Number: Black 4   Sex: F
Group: Black   Fate: (SN) Scheduled Necropsy

Microscopic Findings	
Kidney	Within Normal Limits
Lung	Within Normal Limits
Liver	Extramedullary hematopoiesis, sinusoids, multifocal, mild
Heart	Within Normal Limits
Stomach	Within Normal Limits
Duodenum	Within Normal Limits
Jejunum	Within Normal Limits
Ileum	Within Normal Limits
Cecum	Mineralized cellular debris, crypts, lumen, mutifocal, mild
Colon	Within Normal Limits
Mesenteric Lymph Nodes	Within Normal Limits
Skin (tail base)	Within Normal Limits
Skin (scrotal)	Within Normal Limits

Species: Porcine   Animal Number: Black 5   Sex: M
Group: Black   Fate: (SN) Scheduled Necropsy

Microscopic Findings	
Kidney	Within Normal Limits
Lung	Within Normal Limits
Liver	Extramedullary hematopoiesis, sinusoids, multifocal, mild
Heart	Within Normal Limits
Stomach	Within Normal Limits
Duodenum	Within Normal Limits
Jejunum	Within Normal Limits
Ileum	Within Normal Limits
Cecum X 2	Mineralized cellular debris, crypts, lumen, mutifocal, minimal
Colon	Not Included
Mesenteric Lymph Nodes	Within Normal Limits
Skin (tail base)	Within Normal Limits
Skin (scrotal)	Within Normal Limits

Species: Porcine Animal Number: Black 6 Sex: M
Group: Black Fate: (SN) Scheduled Necropsy

Microscopic Findings	
Kidney	Within Normal Limits
Lung	Within Normal Limits
Liver	Extramedullary hematopoiesis, sinusoids, multifocal, moderate
Heart	Within Normal Limits
Stomach	Within Normal Limits
Duodenum	Within Normal Limits
Jejunum	Within Normal Limits
Ileum	Within Normal Limits
Cecum	Mineralized cellular debris, crypts, lumen, mutifocal, moderate
Colon	Within Normal Limits
Mesenteric Lymph Nodes	Within Normal Limits
Skin (tail base)	Within Normal Limits
Skin (scrotal)	Within Normal Limits

Species: Porcine Animal Number: Black 7 Sex: M
Group: Black Fate: (SN) Scheduled Necropsy

Microscopic Findings	
Kidney	Within Normal Limits
Lung	Erythrocytes, bronchioles and alveoli, locally extensive, mild[2]
Liver	Extramedullary hematopoiesis, sinusoids, multifocal, mild
Heart	Within Normal Limits
Stomach	Within Normal Limits
Duodenum	Within Normal Limits
Jejunum	Within Normal Limits
Ileum	Within Normal Limits
Cecum	Mineralized cellular debris, crypts, lumen, mutifocal, minimal
Colon	Within Normal Limits
Mesenteric Lymph Nodes	Within Normal Limits
Skin (tail base)	Within Normal Limits
Skin (scrotal)	Within Normal Limits

---

[2] Terminal aspiration of blood – not pre-existing death or pathologic, not included in Table 1

Species: Porcine   Animal Number: Black 8   Sex: M
Group: Black   Fate: (SN) Scheduled Necropsy

Microscopic Findings	
Kidney	Within Normal Limits
Lung	Within Normal Limits
Liver	Within Normal Limits
Heart	Within Normal Limits
Stomach	1. Infiltrate, mononuclear cells, lamina propria, multifocal to locally extensive, mild<br>2. Mineralized cellular debris, crypts, lumen, focal, minimal
Duodenum	Within Normal Limits
Jejunum	Within Normal Limits
Ileum	Within Normal Limits
Cecum	Mineralized cellular debris, crypts, lumen, mutifocal, minimal
Colon	Within Normal Limits
Mesenteric Lymph Nodes	Within Normal Limits
Skin (tail base)	Within Normal Limits
Skin (scrotal)	Within Normal Limits

Species: Porcine   Animal Number: Green 1   Sex: F
Group: Green   Fate: (SN) Scheduled Necropsy

Microscopic Findings	
Kidney	Within Normal Limits
Lung	Within Normal Limits
Liver	Extramedullary hematopoiesis, sinusoids, multifocal, moderate
Heart	Not Included
Stomach	Within Normal Limits
Duodenum	Within Normal Limits
Jejunum	Within Normal Limits
Ileum	Within Normal Limits
Cecum	Mineralized cellular debris, crypts, lumen, mutifocal, mild
Colon	Within Normal Limits
Mesenteric Lymph Nodes	Within Normal Limits
Skin (tail base)	Within Normal Limits
Skin (scrotal)	Within Normal Limits

Species: Porcine　　Animal Number: Green 2　　Sex: F
Group: Green　　Fate: (SN) Scheduled Necropsy

Microscopic Findings

Kidney	Within Normal Limits
Lung	Alveolitis, granulomatous, multifocal to locally extensive, mild
Liver	Extramedullary hematopoiesis, sinusoids, multifocal, moderate
Heart	Within Normal Limits
Stomach	Within Normal Limits
Duodenum	Within Normal Limits
Jejunum	Within Normal Limits
Ileum	Within Normal Limits
Cecum	Mineralized cellular debris, crypts, lumen, mutifocal, minimal
Colon	Within Normal Limits
Mesenteric Lymph Nodes	Within Normal Limits
Skin (tail base)	Within Normal Limits
Skin (scrotal)	Within Normal Limits

Species: Porcine　　Animal Number: Green 3　　Sex: F
Group: Green　　Fate: (SN) Scheduled Necropsy

Microscopic Findings

Kidney	Within Normal Limits
Lung	Not Included
Liver	Extramedullary hematopoiesis, sinusoids, multifocal, mild
Heart	Within Normal Limits
Stomach	Within Normal Limits
Duodenum	Within Normal Limits
Jejunum	Within Normal Limits
Ileum	Within Normal Limits
Cecum	Mineralized cellular debris, crypts, lumen, mutifocal, moderate
Colon	Within Normal Limits
Mesenteric Lymph Nodes	Not Included
Skin (tail base)	Within Normal Limits
Skin (scrotal)	Within Normal Limits

Species: Porcine Animal Number: Green 4 Sex: F
Group: Green Fate: (SN) Scheduled Necropsy

Microscopic Findings	
Kidney	Within Normal Limits
Lung	Within Normal Limits
Liver	Extramedullary hematopoiesis, sinusoids, multifocal, mild
Heart	Within Normal Limits
Stomach	Infiltrate, lymphocytes, lamina propria, locally extensive, minimal
Duodenum	Within Normal Limits
Jejunum	Within Normal Limits
Ileum	Within Normal Limits
Cecum	Mineralized cellular debris, crypts, lumen, mutifocal, moderate
Colon	Within Normal Limits
Mesenteric Lymph Nodes	Within Normal Limits
Skin (tail base)	Within Normal Limits
Skin (scrotal)	Within Normal Limits

Species: Porcine Animal Number: Green 5 Sex: M
Group: Green Fate: (SN) Scheduled Necropsy

Microscopic Findings	
Kidney	Within Normal Limits
Lung	Within Normal Limits
Liver	Extramedullary hematopoiesis, sinusoids, multifocal, minimal
Heart	Within Normal Limits
Stomach	Within Normal Limits
Duodenum	Within Normal Limits
Jejunum	Within Normal Limits
Ileum	Within Normal Limits
Cecum	Mineralized cellular debris, crypts, lumen, mutifocal, mild
Colon	Within Normal Limits
Mesenteric Lymph Nodes	Within Normal Limits
Skin (tail base)	Within Normal Limits
Skin (scrotal)	Within Normal Limits

Species: Porcine Animal Number: Green 6 Sex: M
Group: GreenFate: (SN) Scheduled Necropsy

Microscopic Findings

Kidney	Within Normal Limits
Lung	Within Normal Limits
Liver	Extramedullary hematopoiesis, sinusoids, multifocal, moderate
Heart	Within Normal Limits
Stomach	Within Normal Limits
Duodenum	Within Normal Limits
Jejunum	Within Normal Limits
Ileum	Within Normal Limits
Cecum	Mineralized cellular debris, crypts, lumen, mutifocal, minimal
Colon	Within Normal Limits
Mesenteric Lymph Nodes	Within Normal Limits
Skin (tail base)	Within Normal Limits
Skin (scrotal)	Within Normal Limits

Species: Porcine Animal Number: Green 7 Sex: M
Group: Green Fate: (SN) Scheduled Necropsy

Microscopic Findings

Kidney	Within Normal Limits
Lung	Pneumonia, interstitial, lobular distribution, chronic, severe
Liver	Extramedullary hematopoiesis, sinusoids, multifocal, minimal
Heart	Within Normal Limits
Stomach	Within Normal Limits
Duodenum	Within Normal Limits
Jejunum	Within Normal Limits
Ileum	Within Normal Limits
Cecum	Mineralized cellular debris, crypts, lumen, mutifocal, moderate
Colon	Within Normal Limits
Mesenteric Lymph Nodes	Within Normal Limits
Skin (tail base)	Within Normal Limits
Skin (scrotal)	Within Normal Limits

Species: Porcine Animal Number: Green 8 Sex: M
Group: Green Fate: (SN) Scheduled Necropsy

Microscopic Findings

Kidney	Within Normal Limits
Lung	Within Normal Limits
Liver	Not Included
Heart	Within Normal Limits
Stomach	Within Normal Limits
Duodenum	Within Normal Limits
Jejunum	Within Normal Limits
Ileum	Within Normal Limits
Cecum	Mineralized cellular debris, crypts, lumen, mutifocal, minimal
Colon	Within Normal Limits
Mesenteric Lymph Nodes	Within Normal Limits
Skin (tail base)	Within Normal Limits
Skin (scrotal)	Within Normal Limits

DISCUSSION

Samples were received from eight pigs per group with the exception of the purple test group, from which tissues were received from seven pigs. Observations for each test group are listed together in Table 1 and not separated as to sex as, in view of the overall findings, there would be no sex differences in this study.

There were a number of microscopic findings in the animals of this study. The organs having the most common findings were the lungs, liver and cecum. Inflammatory changes were absent from the Blue group, present in lung sections from one animal in each of the Purple, Red and Black groups and in two animals of the Green group. There was no overall similarity or pattern in the reactions that were present. It was concluded that these findings are random occurrences and not test article associated.

Extramedullary hematopoiesis was very common in the livers of test animals of all groups. This is a normal physiologic process in the liver of neonatal piglets and as it was quite evenly spread in intensity and frequency between all of the experimental groups, it was also concluded not to be test article related.

Another very common finding was mineral deposition in the cellular debris present in the crypts of the cecum. This was also present with very similar intensity and frequency in all experimental groups and was concluded to be unrelated to test article exposure. It was likely due to local changes of pH in the deepest parts of the cecal crypts.

Less common was the finding of infiltrates of lymphocytes in the mucosal lamina propria of the stomach. This was a relatively mild and infrequent finding that was seen in a single animal in each group except Red. As such, there was no clear pattern or differentiation in this finding between groups, and it also was concluded to be unrelated to test article exposure.

All other organs were either completely normal (heart, duodenum, jejunum, ileum, colon, mesenteric lymph nodes, tail base skin and scrotal skin) in all animals of all groups or there was one organ (kidney) of one animal in one group that had a minimal spontaneous change.

As there were no patterns or clustering of lesions that would suggest an association with a particular test group or groups and not another group, it was concluded that under the conditions of the experimental protocol, there were no adverse microscopic anatomic effects of the test article.
